# Geographic Variation in Cardiovascular Inflammation among Healthy Women in the Women's Health Study

**DOI:** 10.1371/journal.pone.0027468

**Published:** 2011-11-10

**Authors:** Cheryl R. Clark, Brent Coull, Lisa F. Berkman, Julie E. Buring, Paul M. Ridker

**Affiliations:** 1 Division of General Medicine and Primary Care, Brigham and Women's-Faulkner Hospitalist Program, Harvard Medical School, Boston, Massachusetts, United States of America; 2 Center for Community Health and Health Equity, Brigham and Women's Hospital, Boston, Massachusetts, United States of America; 3 Department of Biostatistics, Harvard School of Public Health, Boston, Massachusetts, United States of America; 4 Harvard Center for Population and Development Studies, Harvard School of Public Health, Boston, Massachusetts, United States of America; 5 Department of Society, Human Development and Health, Harvard School of Public Health, Boston, Massachusetts, United States of America; 6 Division of Preventive Medicine, Brigham and Women's Hospital, Boston, Massachusetts, United States of America; 7 Center for Cardiovascular Disease Prevention, Brigham and Women's Hospital, Boston, Massachusetts, United States of America; 8 Division of Cardiovascular Diseases, Brigham and Women's Hospital, Harvard Medical School, Boston, Massachusetts, United States of America; University of Tor Vergata, Italy

## Abstract

**Background:**

Geographic variation in traditional cardiovascular disease (CVD) risk factors has been observed among women in the US. It is not known whether state-level variation in cardiovascular inflammation exists or could be explained by traditional clinical risk factors and behavioral lifestyle factors.

**Methods and Results:**

We used multilevel linear regression to estimate state-level variation in inflammatory biomarker patterns adjusted for clinical and lifestyle characteristics among 26,029 women free of CVD. Participants derived from the Women's Health Study, a national cohort of healthy middle-aged and older women. Inflammatory biomarker patterns (plasma levels of high-sensitivity C-reactive protein (hsCRP), soluble intercellular adhesion molecule-1 (sICAM-1), and fibrinogen) were compared to state-level patterns of traditional CVD risk factors and global risk scores. We found that all three inflammatory biomarkers exhibited significant state-level variation including hsCRP (lowest vs. highest state median 1.3 mg/L vs. 2.7 mg/L, unadjusted random effect estimate 1^st^ to 99^th^ percentile range for log hsCRP 0.52, p<.001), sICAM-1 (325 ng/ml vs. 366ng/ml, unadjusted random effect estimate 1^st^ to 99^th^ percentile range 0.44, p<.001), and fibrinogen (322 mg/dL vs. 367 mg/dL, unadjusted random effect estimate 1^st^ to 99^th^ percentile range 0.41, p = .001). Neither demographic, clinical or lifestyle characteristics explained away state-level effects in biomarker patterns. Southern and Appalachian states (Arkansas, West Virginia) had the highest inflammatory biomarker values. Regional geographic patterns of traditional CVD risk factors and risk scores did not completely overlap with biomarkers of inflammation.

**Conclusions:**

There is state-level geographic variation in inflammatory biomarkers among otherwise healthy women that cannot be completely attributed to traditional clinical risk factors or lifestyle characteristics. Future research should aim to identify additional factors that may explain geographic variation in biomarkers of inflammation among healthy women.

## Introduction

Inflammation is a major determinant of atherothrombotic cardiovascular disease (CVD) events [1]. Elevated plasma levels of several inflammatory biomarkers including high sensitivity C-reactive protein (hsCRP), soluble intercellular adhesion molecule type 1 (sICAM-1), and fibrinogen have been consistently found to predict future CVD events – including strokes and heart attacks – in healthy populations, particularly in women [1]–[5]. The clinical and public health importance of these inflammatory biomarkers is increasingly recognized [6]–[8]. In a recent comprehensive meta-analysis of 54 prospective cohort studies, the magnitude of risk associated with a one standard deviation increase in hsCRP was at least as large as that associated with a similar change in blood pressure or cholesterol [9].

Despite these data, factors that contribute to population-level differences in cardiovascular inflammation have not been fully explored. From a public health perspective, studying population-level differences in inflammation in healthy populations may be useful to suggest novel prevention strategies that influence atherogenic processes early in the course of CVD. Recently, regional geographic differences in traditional CVD risk factors have been described [10]–[13]. To date, however, comparable data evaluating geographic patterns in inflammatory biomarkers have not been reported. In particular, it is not known whether there is geographic variation in inflammation in relatively lower-risk, healthy populations who have yet to develop CVD. Moreover, it is not clear whether any geographic variation in inflammation would simply reflect the presence of known clinical and behavioral CVD risk factors.

To address these issues, we evaluated state-level profiles of hsCRP, sICAM-1, and fibrinogen in a large-scale cohort of healthy women in the United States, in which the prevalence of traditional CVD risk factors is low. Using multilevel linear regression analysis as well as mapping techniques, we further sought evidence as to (a) whether any observed geographic patterns of inflammation were independent of traditional CVD risk factors (cholesterol, body mass index (BMI), systolic blood pressure, diabetes) and behavioral factors (smoking, exercise, calorie consumption) in these healthy women; and (b) whether inflammatory biomarker geographic patterns share overlap with related CVD risk factors (cholesterol, BMI) and global CVD risk scores (Framingham Point Score, Reynolds Risk Score) in these otherwise healthy women.

## Methods

### Study Participants, Laboratory Evaluation and Survey Data

Written informed consent was obtained from participants for inclusion in the study cohort. Consent procedures and other study procedures for these analyses were reviewed and approved by the Institutional Review Board of Partners HealthCare (Boston, MA).

The study population derived from the 39,876 participants of the Women's Health Study (WHS), a nation-wide study of female health professionals, aged 38 and older, residing in the United States [14]. Participants were free from CVD and cancer at baseline between 1993 and 1996. The WHS cohort included 28,296 women who provided blood samples at baseline. WHS participants eligible for inclusion in this analysis (n = 26,029) had complete baseline data on blood-derived biomarkers and clinical factors, as well as complete data on self-reported demographic, clinical and lifestyle characteristics.

Blood-derived measures included hsCRP, sICAM-1, fibrinogen, total cholesterol, high density lipoprotein cholesterol (HDL-C), low-density lipoprotein cholesterol (LDL-C), and hemoglobin A_1c_ (HbA1c). The laboratory facility that assayed the blood-derived measures was certified by the National Heart, Lung, and Blood Institute/Centers for Disease Control and Prevention Lipid Standardization Program.

Self-reported data assessed participant age, race/ethnicity, height, weight, blood pressure, diabetes, exercise frequency, smoking status, and caloric intake at baseline at the time of the blood draw. The classification of diabetes was further refined using HbA1c of greater or equal to 6.5% to reclassify those who reported not having diabetes as being diabetic, consistent with 2010 American Diabetes Association guidelines. Two global risk scores that predict future CVD events, the Framingham Point Score and the Reynolds Risk Score were calculated with laboratory and clinical survey data (see appendix) [15], [16].

### Statistical Analysis and Geographic Mapping

We report descriptive statistics at the individual level, and percentile ranges at the state-level for demographic characteristics, inflammatory biomarkers, clinical and behavioral risk factors, and global CVD risk score values.

Sequential multilevel regression models were estimated for each outcome measure: three biomarkers (hsCRP, sICAM-1, fibrinogen, assessed separately), two cholesterol measures (total cholesterol, and total cholesterol:HDL-C ratio), BMI, and each global CVD risk score (Framingham Point Score, and Reynolds Risk Score). A “null” model without individual-level covariates was estimated to quantify the total variance at the state level for each outcome measure. An age-adjusted model was estimated to quantify the state-level biomarker variance not accounted for by differences in age distributions within states. A third model with demographic and clinical variables (age, race/ethnicity, diabetes, obesity/overweight, systolic blood pressure, cholesterol) was estimated to quantify the inflammatory biomarker and cholesterol variation between states that was not explained by clinical characteristics of the participants within states. Models with cholesterol measures as the outcome variables did not include cholesterol as covariates. A fourth fully-adjusted set of models was constructed for each of the three inflammatory biomarkers to add the lifestyle factors as covariates (smoking, exercise level, average daily caloric intake). A similar fourth set of models was constructed for cholesterol as outcome measures, also adjusting for clinical and lifestyle factors (and excluding cholesterol as covariates). The measures for BMI (as a continuous variable), the Framingham Point Score and Reynolds Risk Score were not adjusted for covariates. Measurements for hsCRP and BMI were log-transformed prior to incorporation in multivariable models due to skewed values.

Standardized random effect estimates and intraclass correlations at the state level were used to quantify the variance *between* states compared to variance *within* states for each of the three inflammatory biomarkers (hsCRP, sICAM-1, fibrinogen), measures of cholesterol (total cholesterol, total cholesterol:HDL-C ratio), BMI and global risk scores. We estimated state-level random effect coefficients and intraclass correlations using multilevel linear regression in the MIXED procedure in SAS (SAS Institute Inc., Cary, NC, USA).

In multilevel regression analysis, the random effects variance (σ^2^_State_) represents the variance in an outcome variable, in this case in a given biomarker value, attributable to the random factor to which individuals are classified, in this case the state of residence of the participant. State-level standardized random effect estimates are presented as the range between the 1^st^ and 99^th^ percentile of the random effects distribution. Based on the normal assumption for the state-specific random effects, this range corresponds to:
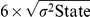



For all multilevel regression models, restricted maximum likelihood estimation (REML) was used to calculate variance components. The statistical significance of the random effects variance component, specifically, the test of the null hypothesis that there is no state-level variation in each measure of inflammation, cholesterol, BMI or global risk score, was assessed by the Wald Z-test in the MIXED procedure in SAS.

Estimates of the state-specific random effects (residuals) for each biomarker, cholesterol measure and risk score outcome variable were mapped within ArcGIS software version 9.3 (ESRI, Redlands, CA) to visualize qualitative differences among the fifty states and the District of Columbia (Washington D.C.) [17]. Five-level color ramps were used to divide the state-specific residuals into quintiles. The map of BMI was presented as state-level median values divided in normal weight (≤ 24.9 kg/m2) and overweight categories (25.0 kg/m2 and over) to facilitate comparison with published maps. Last, multilevel regression models were repeated using the ten Federal Standard Regions as the random classification variable to test for the presence of broad regional patterns in the data.

All statistical tests were computed in SAS 9.2 (SAS Institute Inc., Cary, NC). Maps were created in ArcGIS ArcMAP software version 9.3 (ESRI, Redlands, CA).

## Results

The WHS cohort is geographically diverse. All states except Hawaii (n = 8) and Washington D.C. (n = 25) were well-sampled. In all other states there were more than fifty WHS participants per state, ranging from n = 58 WHS participants in Alaska to n = 2,639 WHS participants in California.

Table 1 presents biomarker levels, clinical and lifestyle data on all study participants, as well as state-level distributions of these characteristics, from the 1^st^ to the 99^th^ percentile. Among states that were well-sampled, the full distribution of median hsCRP values had a clinically significant range of moderately-low risk values (1.3 to 1.6 mg/L in Iowa, Massachusetts, and New York) to moderately-higher risk values (from 2.5 to 2.7 mg/L in Texas, Louisiana, Alabama, Arkansas and West Virginia). The lowest state-level values for both sICAM-1 (325 ng/ml) and fibrinogen (322 mg/dL) were seen in Iowa. The highest state-level values were seen in Arkansas for both sICAM-1 (366 ng/ml) and fibrinogen (367 mg/dL). State-level median biomarker values are listed in Table S1.

**Table 1:** Selected Demographic, Lifestyle, and Clinical Characteristics of Women's Health Study Participants: With State-Level Comparisons.

	All Participants	U.S. State-Level Percentile Ranges				
	N = 26,029	N = 51				
	All States	1^st^	25^th^	50^th^	75^th^	99^th^
**Demographic**						
Age, median (IQR), y	53 (49- 59)	51	52	53	53	54
NH-White†	24,716 (95.0)	84%	93%	96%	98%	100%
Non-White	1,313 (5.0)	16%	7%	4%	2%	0%
NH-Black	444 (1.7)	-	-	-	-	-
Hispanic	225 (0.9)	-	-	-	-	-
Asian Pacific	343 (1.3)	-	-	-	-	-
Other/Unknown	301 (1.1)	-	-	-	-	-
**Lifestyle**						
Current smoker	2,983 (11)	4%	9%	11%	13%	17%
Exercise rarely/never	9,642 (37)	16%	33%	38%	40%	52%
Median Daily Caloric intake,	1676 (1350- 2052)	1354	1646	1688	1721	1946
median (IQR), kCal						
**Clinical**						
hsCRP median (IQR), mg/L	2.0 (0.80- 4.3)	0.9	1.8	2.1	2.2	2.7
sICAM-1 median (IQR), ng/mL	342 (301- 394)	320	338	344	349	366
Fibrinogen median (IQR), mg/dL	351 (307- 403)	322	345	351	356	385
Total Cholesterol, median (IQR), mg/dL	208 (184- 235)	197	206	208	210	218
Cholesterol:HDL-C Ratio, median (IQR)	4.0 (3.2- 4.9)	3.5	3.9	4.0	4.1	4.3
Framingham Point Score, median (IQR)	12 (9- 15)	11	12	12	12	13
Reynolds Risk Score, median (IQR)	1.5 (0.74- 3.2)	0.9	1.3	1.4	1.6	2.0
BMI, median (IQR), kg/m2	24.9 (22.5- 28.3)	23.3	24.6	24.9	25.1	25.8
Systolic blood pressure category,	125 (115- 135)	115	125	125	125	125
median (IQR), mmHg						
Percent with diabetes	754 (2.9)	0%	2.4%	2.8%	3.5%	4.7%

Source: Women's Health Study (WHS). Figures are No.(%) unless otherwise noted. Abbreviations: NH (Non-Hispanic ethnicity); hsCRP (high-sensitivity C-reactive protein); sICAM-1 (soluble intercellular adhesion molecule); HDL-C (high-density lipoprotein cholesterol); BMI (body mass index); IQR (inter-quartile range). State-level percentiles based on ranges of 50 US States plus the District of Columbia. “Asian” includes Asian/Pacific Islander; “Other/Unknown,” includes American Indian/Native American/Alaska Native.

Among the traditional risk factors, median total cholesterol values were clinically low risk to borderline-elevated, lowest in Iowa and Utah (197 mg/dL) and highest in Wyoming (218 mg/dL) and Arkansas (215 mg/dL). For states that were well-sampled, median values for the total cholesterol:HDL-C ratio were moderately-low risk values from 3.7 (California) to 4.3 (West Virginia). Among half of states (n = 25), median BMI values were in the overweight range (24.95 – 25.8 kg/m^2^) highest in Utah and West Virginia. No state had a median BMI value in the obese range among WHS participants. Framingham Point Score values ranged from a median value of 11 (including Delaware and Iowa), corresponding to 1% 10-year risk of CVD events, to 13 (Arkansas and Pennsylvania), corresponding to a 2% 10-year risk of CVD events. Similarly, Reynolds Risk Score median values ranged from a 1.2% 10-year risk of CVD events (Vermont, Maryland, Iowa, Oregon, Nebraska), to 2.0% 10-year risk (Arkansas).

**Figure 1:**
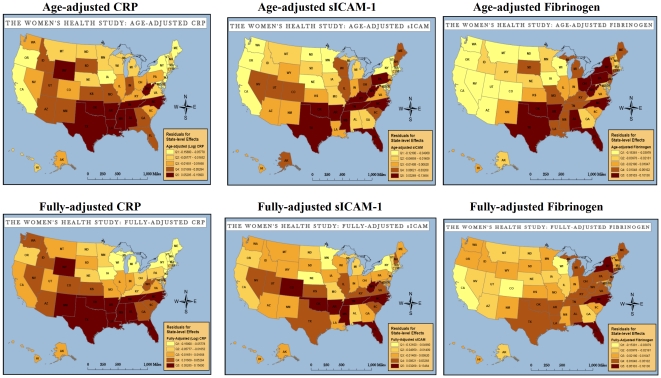
State-level Variation in Cardiovascular Biomarkers of Inflammation in the Women's Health Study. Source: Women's Health Study Participants. Abbreviations: Q1: Lowest quintile of inflammation; Q5: Highest quintile of inflammation. Fully-adjusted models include: age, race/ethnicity, obese/overweight status, systolic blood pressure, diabetes status, HDL-C, LDL-C, smoking, exercise, caloric intake.

Figure 1 shows state-level patterns in biomarkers of inflammation adjusted for (a) age, and (b) for the clinical and lifestyle characteristics of women living in the state. State-level random effect estimates quantifying the biomarker patterns are listed in Table 2. Unadjusted state-level intraclass correlations ranged between 0.35% and 0.76%. Statistically significant state-level variation was seen for each of the three biomarkers before and after adjustment for covariates. State-level patterns in hsCRP were only marginally affected by adjustment for clinical and lifestyle characteristics of WHS participants. For hsCRP, the unadjusted state-level variance was 0.52 (p<.001). Adjustment for clinical and lifestyle characteristics decreased state-level variation in hsCRP by 12%. The presence of clinical factors (overweight and obesity, systolic blood pressure, diabetes, HDL-C, LDL-C) appeared to explain more state-level variation in hsCRP than lifestyle factors (exercise, smoking status, daily caloric intake), though statistically significant state-level variation in hsCRP was still apparent after full adjustment for the above covariates (0.46, p<.001). More of the state-level variance in sICAM-1 and fibrinogen was explained by clinical and lifestyle characteristics than was seen for hsCRP. In particular, state-level variance in both sICAM-1 and fibrinogen were more sensitive to women's lifestyle characteristics than seen in hsCRP. The linear associations between biomarkers of inflammation and study participants' clinical and lifestyle characteristics as estimated in multilevel models are presented in Tables S2, S3 and S4.

**Table 2:** State-Level Variation in Cardiovascular Inflammatory Biomarkers, Risk Factors, and Clinical Risk Scores.

		State-level Random Effects Estimates (P Value)			
Outcome Variable	Value	Model I	Model II	Model III	Model IV
hsCRP(Log)					
	Estimate* (P value)	0.52 (<.001)	0.50 (<.001)	0.47 (<.001)	0.46 (<.001)
	ICC (%)	0.76	0.71	0.78	0.76
sICAM-1					
	Estimate* (P value)	0.44 (<.001)	0.42 (.001)	0.31 (.01)	0.26 (.01)
	ICC (%)	0.55	0.50	0.30	0.23
Fibrinogen					
	Estimate* (P value)	0.41 (.001)	0.41 (.001)	0.30 (0.005)	0.26 (.01)
	ICC (%)	0.47	0.48	0.29	0.22
Total Cholesterol†					
	Estimate* (P value)	0.43 (<.001)	0.44 (<.001)	0.45 (.001) †	0.44 (.001)†
	ICC (%)	0.52	0.55	0.58	0.56
Total Cholesterol: HDL-C Ratio†					
	Estimate* (P value)	0.47 (<.001)	0.46 (<.001)	0.40 (<.001) †	0.34 (.001)†
	ICC (%)	0.61	0.60	0.52	0.40
BMI(Log)					
	Estimate* (P value)	0.35 (.001)	-	-	-
	ICC (%)	0.35			
Framingham Point Score					
	Estimate* (P value)	0.40 (<.001)	-	-	-
	ICC (%)	0.45			
Reynolds Risk Score					
	Estimate* (P value)	0.37 (.001)	-	-	-
	ICC (%)	0.38			

Source: Women's Health Study. Abbreviations: hsCRP(Log) (natural log of high sensitivity C-reactive protein);

sICAM-1 (soluble intercelluar adhesion molecule); HDL-C (high-density lipoprotein cholesterol); LDL-C (low-density lipoprotein cholesterol); BMI(Log) (natural log of body mass index); ICC (intra-class correlation).

Multilevel linear regression random effect estimates and intraclass correlations estimate the presence non-zero variance in inflammatory biomarkers, cholesterol, BMI (log), or risk scores at the state level. P value ≤ 0.05 rejects null hypothesis of zero variance among states.

*Estimates presented as 1^st^ to 99^th^ percentile range.

†Models of cholesterol as the outcome variable do not control for cholesterol measures within the model. Models for BMI, Framingham and Reynolds risk scores are not adjusted for covariates.

Model I. Unadjusted.

Model II. Age-adjusted.

Model III. Adjusted for age, race, obesity/overweight, diabetes, systolic blood pressure, HDL-C^†^, LDL-C^†^

Model IV. Adjusted for age, race, obesity/overweight, diabetes, systolic blood pressure, HDL-C^†^, LDL-C^†^, exercise,

smoking status, daily caloric intake.

**Figure 2:**
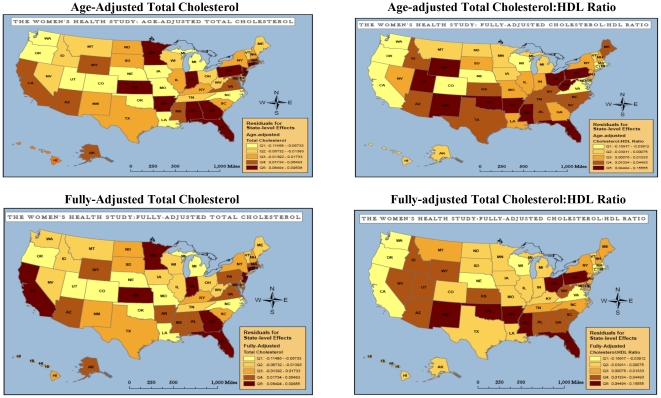
State-level Variation in Cholesterol in the Women's Health Study. Source: Women's Health Study Participants. Abbreviations: Q1: Lowest quintile of risk; Q5: Highest quintile of risk. Fully-adjusted models include: age, race/ethnicity, obese/overweight status, systolic blood pressure, diabetes status, smoking, exercise, caloric intake.

**Figure 3:**
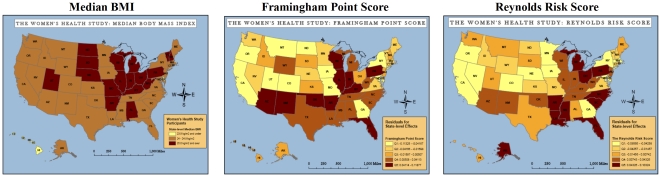
State-level Variation in Cardiovascular Risk Scores and State Median BMI in the Women's Health Study. Source: Women's Health Study Participants. Abbreviations: Q1: Lowest quintile of risk; Q5: Highest quintile of risk. Models not adjusted for covariates.

Figure 2 shows comparable state-level patterns of geographic variation in total cholesterol, and the total cholesterol:HDL-C ratio. The total cholesterol:HDL-C ratio can be interpreted as the proportion of the cholesterol profile that is made up of HDL-C or “good” cholesterol. State-level variance in total cholesterol was not explained by individual-level clinical and lifestyle characteristics (Table 2). In contrast, state-level variance in the total cholesterol:HDL-C ratio was reduced by 28% after full adjustment for clinical and lifestyle covariates. Figure 3 displays state-level median BMI, and state-level variation in the Framingham Point Score, and the Reynolds Risk Score. State-level variations in BMI, the Framingham Point Score, and Reynolds Risk Score were statistically significant as shown in Table 2. Full size figures of biomarker and risk score maps are available as online supplements (Figures S1, S2, S3, S4, S5, S6, S7, S8, S9, S10, S11, S12, S13).

Statistically significant broad regional patterns exist for all biomarkers and clinical risk factors (Table 3). Almost all indicators identified states in the southeastern and Appalachian Standard Federal Regions IV and VI as areas of highest risk. After adjustment for covariates, regional patterns for hsCRP and sICAM-1 were similar; highest levels of inflammation were seen in Regions IV and VI. Regions IV and VI and mid-Atlantic Region II (New York and New Jersey) had the highest levels of fibrinogen. Similar to hsCRP, sICAM-1 and fibrinogen, the total cholesterol to HDL-C ratio, and the Framingham and Reynolds Risk Scores identified Regions IV and VI as higher risk areas. Both global CVD risk scores also identified the mid-western Region V as an area of higher risk for future CVD events. Regional inflammatory biomarker patterns differed from patterns of BMI and total cholesterol without adjustment for HDL-C. Region IX was identified as having the highest total cholesterol values. BMI was highest in Regions V and VII. The areas of lowest risk differed for each marker and risk factor, and most commonly included Region II (hsCRP, sICAM-1 and Reynolds Risk Score), Region VIII (fibrinogen, total cholesterol, Framingham Point Score, and Reynolds Risk Score), Region IX (fibrinogen, total cholesterol to HDL-C ratio, and BMI), and Region X (total cholesterol, total cholesterol to HDL-C ratio, and Framingham Point Score).

**Table 3:** Federal Regional-level Variation in Cardiovascular Inflammation, Cholesterol, BMI and Cardiovascular Risk Scores.

	Random effects Estimate§	Intraclass correlation (%)	P Value	Highest risk	Lowest risk
hsCRP(Log) *	0.49	0.85	0.02	Region IV, VI	Region I, II
sICAM-1*	0.21	0.15	0.05	Region IV, VI	Region II
Fibrinogen*	0.30	0.31	0.04	Region II, IV, VI	Region VIII, IX
Cholesterol†	0.26	0.19	0.05	Region IX	Region VIII, X
Cholesterol:HDL-C ratio†	0.27	0.25	0.04	Region IV, VI	Region IX, X
BMI(Log)‡	0.32	0.28	0.03	Region V, VII	Region IX
Framingham Point Score‡	0.30	0.26	0.04	Region IV, V, VI	Region VIII, X
Reynolds Risk Score‡	0.25	0.18	0.04	Region IV, V, VI	Region II, VII, VIII

Source: Women's Health Study.

*Biomarker models adjust for age, race, obesity/overweight, diabetes, systolic blood pressure, HDL-C, LDL-C, smoking, exercise, daily caloric intake.

† Models of total cholesterol and cholesterol:HDL-C ratio adjust for age, race, obesity/overweight, diabetes, systolic blood pressure, smoking, exercise, daily caloric intake.

‡ BMI, Framingham Point Score and Reynolds Risk Score do not include covariates.

§ Estimates presented as 1^st^ to 99^th^ percentile range.

U.S. Standard Federal Regions:

Region I: Connecticut, Maine, Massachusetts, New Hampshire, Rhode Island, Vermont.

Region II: New Jersey, New York.

Region III: Delaware, District of Columbia, Maryland, Pennsylvania, Virginia, West Virginia.

Region IV: Alabama, Florida, Georgia, Kentucky, Mississippi, North Carolina, South Carolina, Tennessee.

Region V: Illinois, Indiana, Michigan, Minnesota, Ohio, Wisconsin.

Region VI: Arkansas, Louisiana, New Mexico, Oklahoma, Texas.

Region VII: Iowa, Kansas, Missouri, Nebraska.

Region VIII: Colorado, Montana, North Dakota, South Dakota, Utah, Wyoming.

Region IX: Arizona, California, Hawaii, Nevada.

Region X: Alaska, Idaho, Oregon, Washington.

## Discussion

Our analysis of 26,029 healthy women across the United States found significant state to state variation in inflammation, as measured by hsCRP, sICAM-1 and fibrinogen. We found that the three biomarkers of inflammation identified similar patterns of variability. Iowa was most consistently identified as having the lowest median level of inflammation among healthy women. States in the southeast and Appalachia, namely, Arkansas and West Virginia, were most likely to have elevated levels of inflammation. Notably, the range of state-level variation we observed was clinically relevant. For hsCRP in particular, state-level median values ranged from lower risk values (1.3 mg/L in Iowa) to moderately-higher risk values (2.7 mg/L in West Virginia). The southeast was identified as the area of highest risk by each biomarker of inflammation, global risk score, and the total cholesterol:HDL-C ratio. Moreover, none of the clinical and lifestyle measures of overweight and obesity, diabetes, systolic blood pressure, HDL-C, LDL-C, smoking status, exercise patterns, or caloric intake fully accounted for the state-level variation in biomarkers we observed. More variability in sICAM-1 and fibrinogen was accounted for by clinical and lifestyle factors than was seen for hsCRP, though state-level patterns in all three biomarkers were still apparent after adjustment for covariates.

Our data are consistent with findings in women from the Reasons for Geographic and Racial Differences in Stroke (REGARDS) study that found elevated hsCRP levels in the “stroke belt” region of the US (Alabama, Arkansas, Georgia, Louisiana, Mississippi, North Carolina, South Carolina, and Tennessee) [18]. Women in the REGARDS study who resided in the stroke-belt region had 20% higher odds of an elevated hsCRP of > 3 mg/L than women in the non-stroke belt regions; this risk was independent of race/ethnicity, smoking status, cholesterol status, overweight/obesity, blood pressure or the presence of diabetes. Similarly, El Saed et al. found geographic variability in stroke incidence in the Cardiovascular Health Study [19]. As in our data, these differences in stroke incidence between high and low risk geographies in the US were not explained by differences in traditional CVD risk factors or medication use [20]. Our maps show a geographic pattern of inflammation that is generally consistent with CVD mortality maps from the Centers of Disease Control and Prevention (CDC) that show the southeastern US as an area of high CVD mortality risk among women. Our fibrinogen map appeared to match a west to east gradient in risk, consistent with the known geography of CVD mortality.

The causal patterns underlying these regional effects are uncertain. One potential cause is suggested in epidemiologic data that demonstrate an environmental effect of particulate matter exposure and elevated levels of fibrinogen and hsCRP in select populations [21]. In other studies, populations in rural and impoverished areas have been found to be at higher risk of mortality than more urban, wealthy areas [22]. Our results suggest the need for additional studies that tease out the effects of state-level characteristics or contextual factors that may be associated with geographically distributed risk for elevated inflammation.

Limitations of our study merit attention. One limitation is that WHS participants are chiefly healthy, non-obese, of white non-Hispanic race/ethnicity, and are health care professionals. Thus, while internally valid, studies of other demographic groups are needed prior to generalizing these data. These characteristics of the WHS likely truncate and therefore under-estimate variability that may exist among states that are more diverse than the WHS population. However, the strength of examining geographic variation in this healthy cohort is its applicability to primary prevention. Our data highlight the extent to which inflammation varies geographically among women prior to the development of clinically apparent CVD. Inflammation is known to correlate with future risk of heart disease among women, and biomarkers of inflammation improve risk prediction for future CVD events [23]. Our study suggests the need to understand population-level contributors to geographic patterns in the early development of CVD among women, above and beyond differences in the prevalence of known traditional risk factors.

Another limitation, our study is cross-sectional and only measures inflammatory markers at one point in time during middle and older age. With our study design, we cannot draw causal associations between early life state of residence and inflammation among individual women. An emerging literature suggests that those who have lived in the southeastern US in childhood or adolescence have an elevated risk for an incident CVD event, even if they had subsequently left these regions [24]. In the WHS cohort, elevated inflammation in women measured at older ages is known to correlate with subsequent CVD events [2]. Thus, the extent to which geographic exposures are correlated with risk of elevated inflammation, population-health prevention efforts should address these exposures across the life-course.

In summary, our study contributes to the literature on population health by demonstrating geographic variability in inflammation among healthy women. Even among otherwise healthy, chiefly non-Hispanic white female health professionals, we found that those living in southern and Appalachian states had high levels of inflammation compared to women in other geographies. The geographic variation in inflammation we observed was in excess of traditional clinical risk factors and lifestyle factors – a pattern that has been observed previously in stroke outcomes [25]. Our findings suggest a need for additional research to understand why there is geographic variation in risk in these early biomarkers of atherosclerosis to inform primary prevention strategies. Importantly, further research should find novel population-based strategies to address the increased risk observed in the southern and Appalachian US, in ways that complement the control of traditional risk factors.

## Supporting Information

Figure S1 **State-level Variation in Age-Adjusted C-reactive Protein (CRP) in the Women's Health Study.** Source: Women's Health Study Participants. Abbreviations: Q1: Lowest quintile of inflammation; Q5: Highest quintile of inflammation.
(TIF)

Figure S2 **State-level Variation in Age-Adjusted sICAM-1 in the Women's Health Study.** Source: Women's Health Study Participants. Abbreviations: Q1: Lowest quintile of inflammation; Q5: Highest quintile of inflammation.
(TIF)

Figure S3 **State-level Variation in Age-Adjusted Fibrinogen in the Women's Health Study.** Source: Women's Health Study Participants. Abbreviations: Q1: Lowest quintile of inflammation; Q5: Highest quintile of inflammation.
(TIF)

Figure S4 **State-level Variation in Fully-Adjusted C-reactive Protein (CRP) in the Women's Health Study.** Source: Women's Health Study Participants. Abbreviations: Q1: Lowest quintile of inflammation; Q5: Highest quintile of inflammation. Fully-adjusted models include: age, race/ethnicity, obese/overweight status, systolic blood pressure, diabetes status, HDL-C, LDL-C, smoking, exercise, caloric intake.
(TIF)

Figure S5 **State-level Variation in Fully-Adjusted sICAM-1 in the Women's Health Study.** Source: Women's Health Study Participants. Abbreviations: Q1: Lowest quintile of inflammation; Q5: Highest quintile of inflammation. Fully-adjusted models include: age, race/ethnicity, obese/overweight status, systolic blood pressure, diabetes status, HDL-C, LDL-C, smoking, exercise, caloric intake.
(TIF)

Figure S6 **State-level Variation in Fully-Adjusted Fibrinogen in the Women's Health Study.** Source: Women's Health Study Participants. Abbreviations: Q1: Lowest quintile of inflammation; Q5: Highest quintile of inflammation. Fully-adjusted models include: age, race/ethnicity, obese/overweight status, systolic blood pressure, diabetes status, HDL-C, LDL-C, smoking, exercise, caloric intake.
(TIF)

Figure S7 **State-level Variation in Age-Adjusted Total Cholesterol in the Women's Health Study.** Source: Women's Health Study Participants. Abbreviations: Q1: Lowest quintile of risk; Q5: Highest quintile of risk.
(TIF)

Figure S8 **State-level Variation in Age-Adjusted Total Cholesterol:HDL Ratio in the Women's Health Study.** Source: Women's Health Study Participants. Abbreviations: Q1: Lowest quintile of risk; Q5: Highest quintile of risk.
(TIF)

Figure S9 **State-level Variation in Fully-Adjusted Total Cholesterol in the Women's Health Study.** Source: Women's Health Study Participants. Abbreviations: Q1: Lowest quintile of risk; Q5: Highest quintile of risk. Fully-adjusted models include: age, race/ethnicity, obese/overweight status, systolic blood pressure, diabetes status, smoking, exercise, caloric intake.
(TIF)

Figure S10 **State-level Variation in Fully-Adjusted Total Cholesterol:HDL Ratio in the Women's Health Study.** Source: Women's Health Study Participants. Abbreviations: Q1: Lowest quintile of risk; Q5: Highest quintile of risk. Fully-adjusted models include: age, race/ethnicity, obese/overweight status, systolic blood pressure, diabetes status, smoking, exercise, caloric intake.
(TIF)

Figure S11 **State-level Variation in Median Body Mass Index in the Women's Health Study.** Source: Women's Health Study Participants. Model not adjusted for covariates.
(TIF)

Figure S12 **State-level Variation in the Framingham Point Score in the Women's Health Study.** Source: Women's Health Study Participants. Abbreviations: Q1: Lowest quintile of risk; Q5: Highest quintile of risk. Model not adjusted for covariates.
(TIF)

Figure S13 **State-level Variation in the Reynolds Risk Score in the Women's Health Study.** Source: Women's Health Study Participants. Abbreviations: Q1: Lowest quintile of risk; Q5: Highest quintile of risk. Model not adjusted for covariates.
(TIF)

Table S1 **State-Level Median Values for Biomarkers of Inflammation among Healthy Women in the Women's Health Study (N = 26,029).** *Estimates for District of Columbia and Hawaii based on fewer than 50 participants.
(DOC)

Table S2 **Fully-Adjusted Multi-Level Linear Regression Models on log(hsCRP): Standardized β Coefficients, 95% Confidence Intervals and Wald Tests (N = 26,029).** Source: Women's Health Study (WHS). Abbreviations: log(hsCRP) log (High-Sensitivity C-Reactive Protein); NH (Non-Hispanic ethnicity); HDL-C (high-density lipoprotein cholesterol); LDL-C (low-density lipoprotein cholesterol); BMI (body mass index); ref. (reference category). Standardized β coefficients estimated via multi-level linear regression models adjusted for listed covariates. In our primary analysis race ethnicity is modeled as non-White vs. NH-White due to small numbers. In models with race as a categorical variable, log(hsCRP) is lower among Asian/Pacific Islanders compared to NH-Whites [Std β -0.26; 95% CI -0.35, -0.16; *P* Value < 0.0001]. Hispanics tended to have higher log(hsCRP) compared to NH-Whites [Std β 0.04; 95% CI -0.07, 0.16; *P* Value 0.48]. NH-Blacks tended to have higher log(hsCRP) compared to NH-Whites [Std β 0.02; 95% CI -0.06, 0.11; *P* Value 0.61].
(DOC)

Table S3 **Fully-Adjusted Multi-Level Linear Regression Models on sICAM-1: Standardized β Coefficients, 95% Confidence Intervals and Wald Tests (N = 26,029).** Source: Women's Health Study (WHS). Abbreviations: NH (Non-Hispanic ethnicity); HDL-C (high-density lipoprotein cholesterol); LDL-C (low-density lipoprotein cholesterol); BMI (body mass index); ref (reference category). Standardized β coefficients estimated via multi-level linear regression models adjusted for listed covariates. In our primary analysis race ethnicity is modeled as non-White vs. NH-White due to small numbers. In models with race as a categorical variable, sICAM-1 is lower among Asian/Pacific Islanders compared to NH-Whites [Std β -0.31; 95% CI -0.41, -0.22; *P* Value < 0.0001]. Hispanics tended to have higher sICAM-1 compared to NH-Whites [Std β 0.02; 95% CI -0.10, 0.14; *P* Value 0.72]. NH-Blacks tended to have lower sICAM-1 values compared to NH-Whites [Std β -0.67; 95% CI -0.76, 0.59; *P* Value < 0.0001].
(DOC)

Table S4 **Fully-Adjusted Multi-Level Linear Regression Models on Fibrinogen: Standardized β Coefficients, 95% Confidence Intervals and Wald Tests (N = 26,029).** Source: Women's Health Study (WHS). Abbreviations: NH (Non-Hispanic ethnicity); HDL-C (high-density lipoprotein cholesterol); LDL-C (low-density lipoprotein cholesterol); BMI (body mass index). Standardized β coefficients estimated via multi-level linear regression models adjusted for listed covariates. In our primary analysis race ethnicity is modeled as non-White vs. NH-White due to small numbers. In models with race as a categorical variable, fibrinogen is higher among Asian/Pacific Islanders compared to NH-White [Std β 0.07; 95% CI -0.03, 0.17; *P* Value 0.17]. Hispanics tended to have higher fibrinogen levels compared to NH-Whites [Std β 0.09; 95% CI -0.03, 0.21; *P* Value 0.15]. NH-Blacks tended to have higher fibrinogen levels compared to NH-Whites [Std β 0.42; 95% CI 0.33, 0.50; *P* Value < 0.0001].
(DOC)
