# Supplementary figures and images for: Geographic Variation in Cardiovascular Inflammation among Healthy Women in the Women's Health Study
Source: PLoS One. 2011 Nov 10;6(11):e27468. doi: 10.1371/journal.pone.0027468 (PMC3213140; doi:10.1371/journal.pone.0027468)

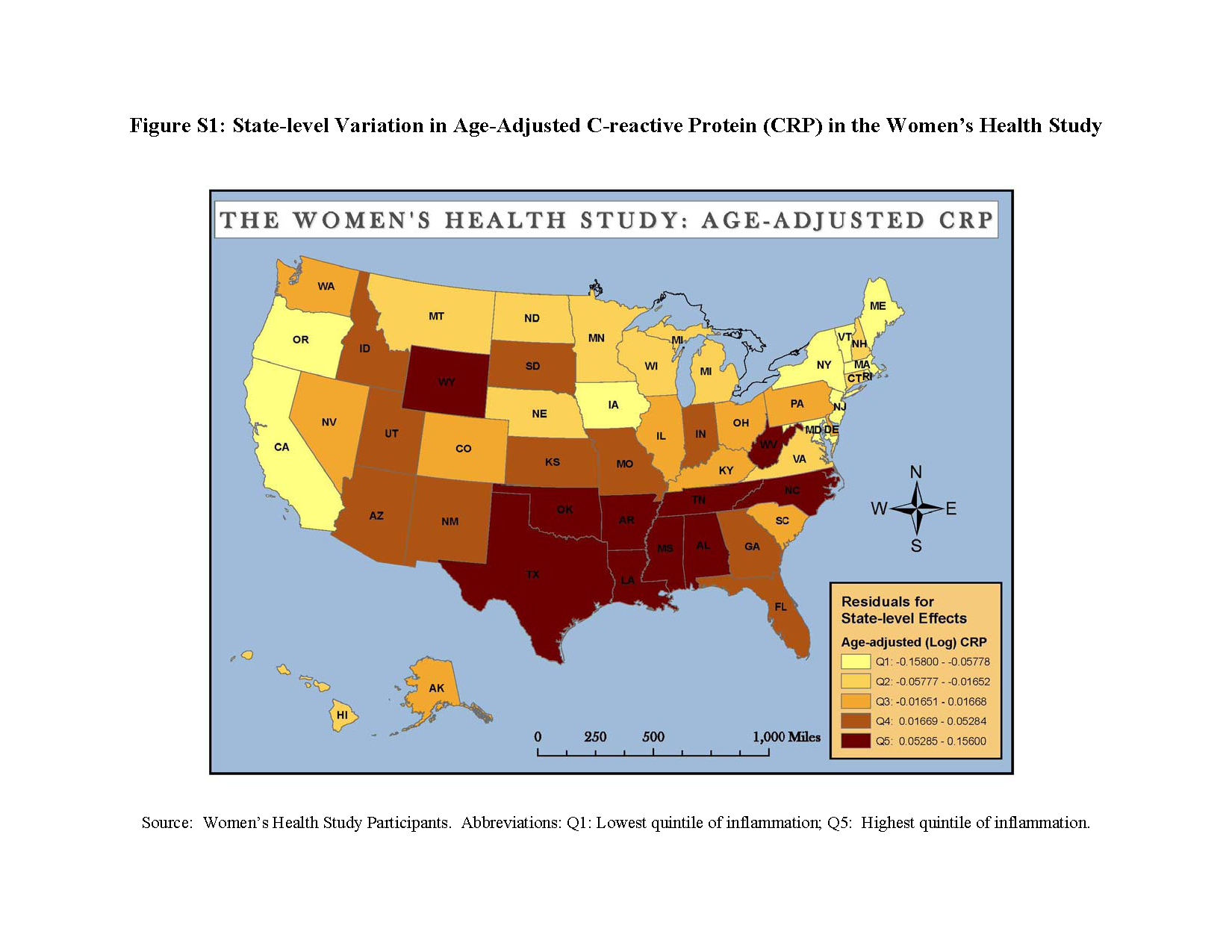

Supplement: Figure S1 — State-level Variation in Age-Adjusted C-reactive Protein (CRP) in the Women's Health Study. Source: Women's Health Study Participants. Abbreviations: Q1: Lowest quintile of inflammation; Q5: Highest quintile of inflammation. (TIF) [file pone.0027468.s001.tif]

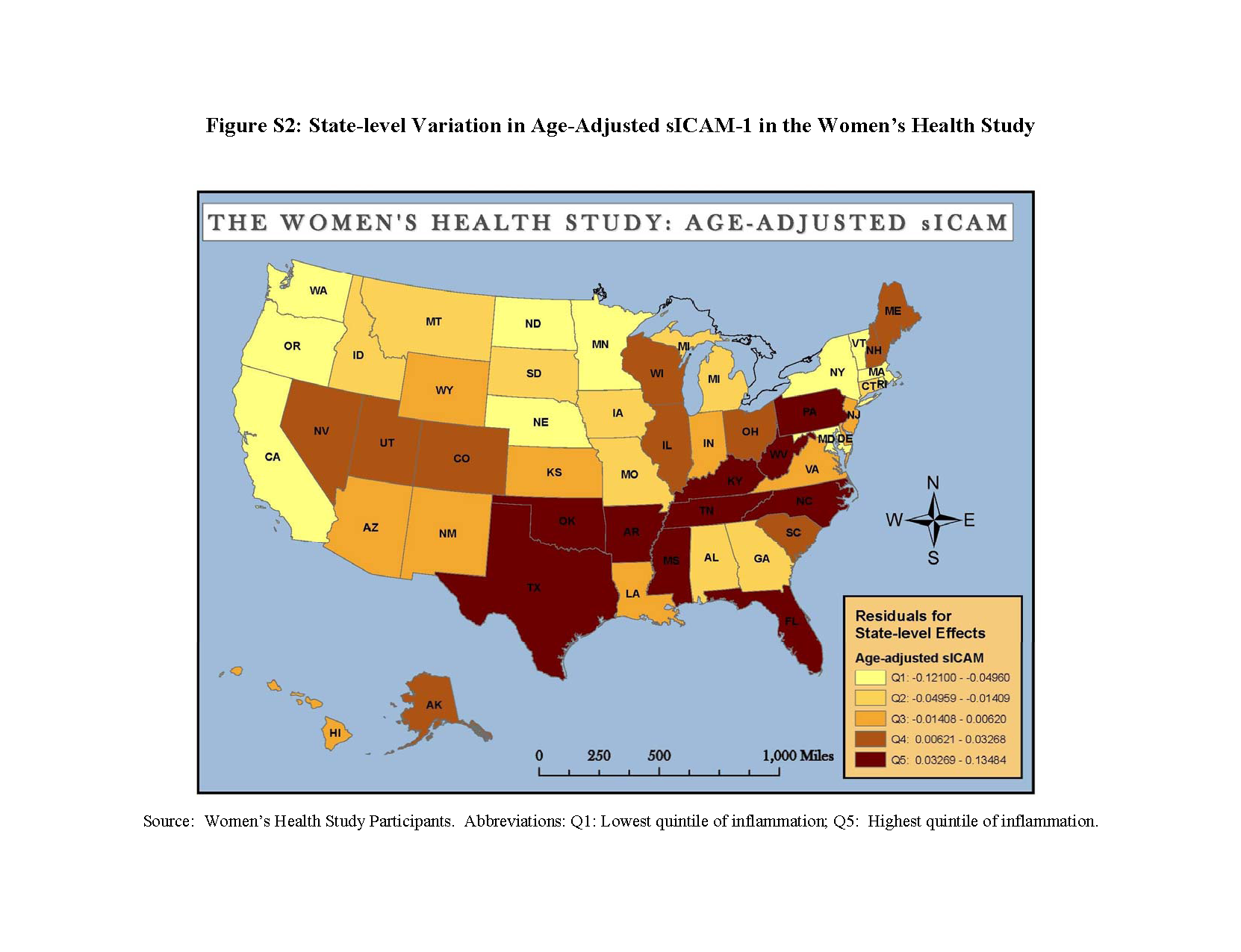

Supplement: Figure S2 — State-level Variation in Age-Adjusted sICAM-1 in the Women's Health Study. Source: Women's Health Study Participants. Abbreviations: Q1: Lowest quintile of inflammation; Q5: Highest quintile of inflammation. (TIF) [file pone.0027468.s002.tif]

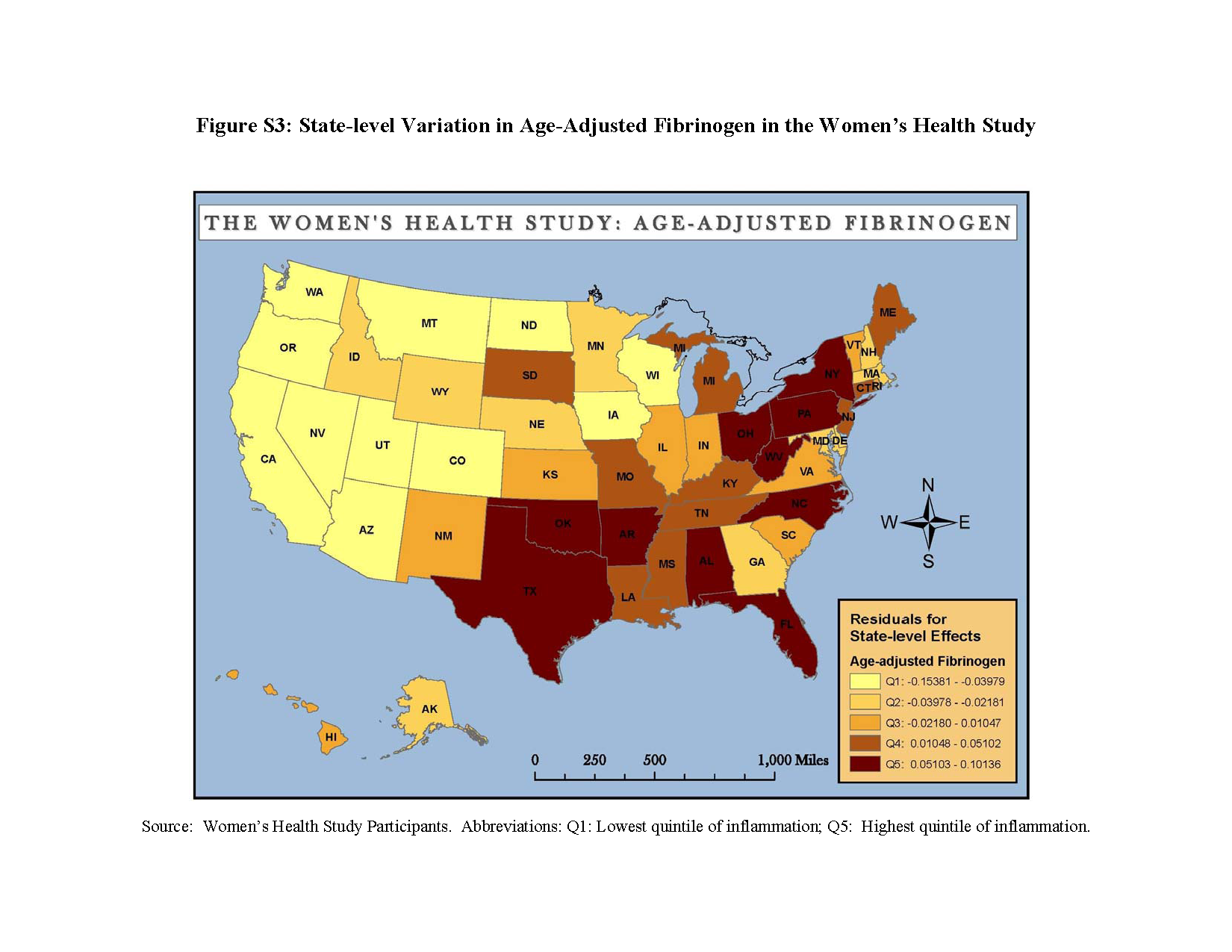

Supplement: Figure S3 — State-level Variation in Age-Adjusted Fibrinogen in the Women's Health Study. Source: Women's Health Study Participants. Abbreviations: Q1: Lowest quintile of inflammation; Q5: Highest quintile of inflammation. (TIF) [file pone.0027468.s003.tif]

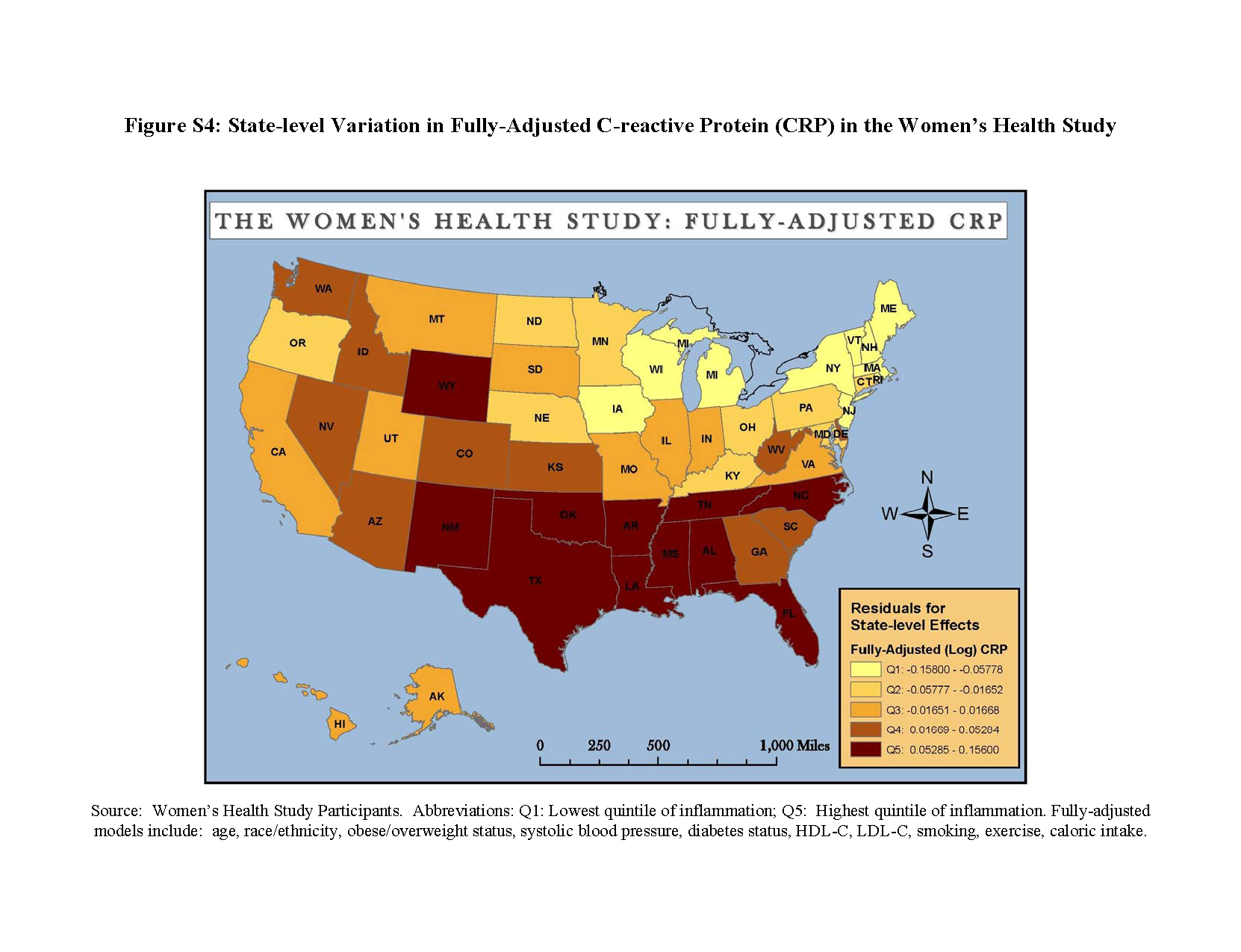

Supplement: Figure S4 — State-level Variation in Fully-Adjusted C-reactive Protein (CRP) in the Women's Health Study. Source: Women's Health Study Participants. Abbreviations: Q1: Lowest quintile of inflammation; Q5: Highest quintile of inflammation. Fully-adjusted models include: age, race/ethnicity, obese/overweight status, systolic blood pressure, diabetes status, HDL-C, LDL-C, smoking, exercise, caloric intake. (TIF) [file pone.0027468.s004.tif]

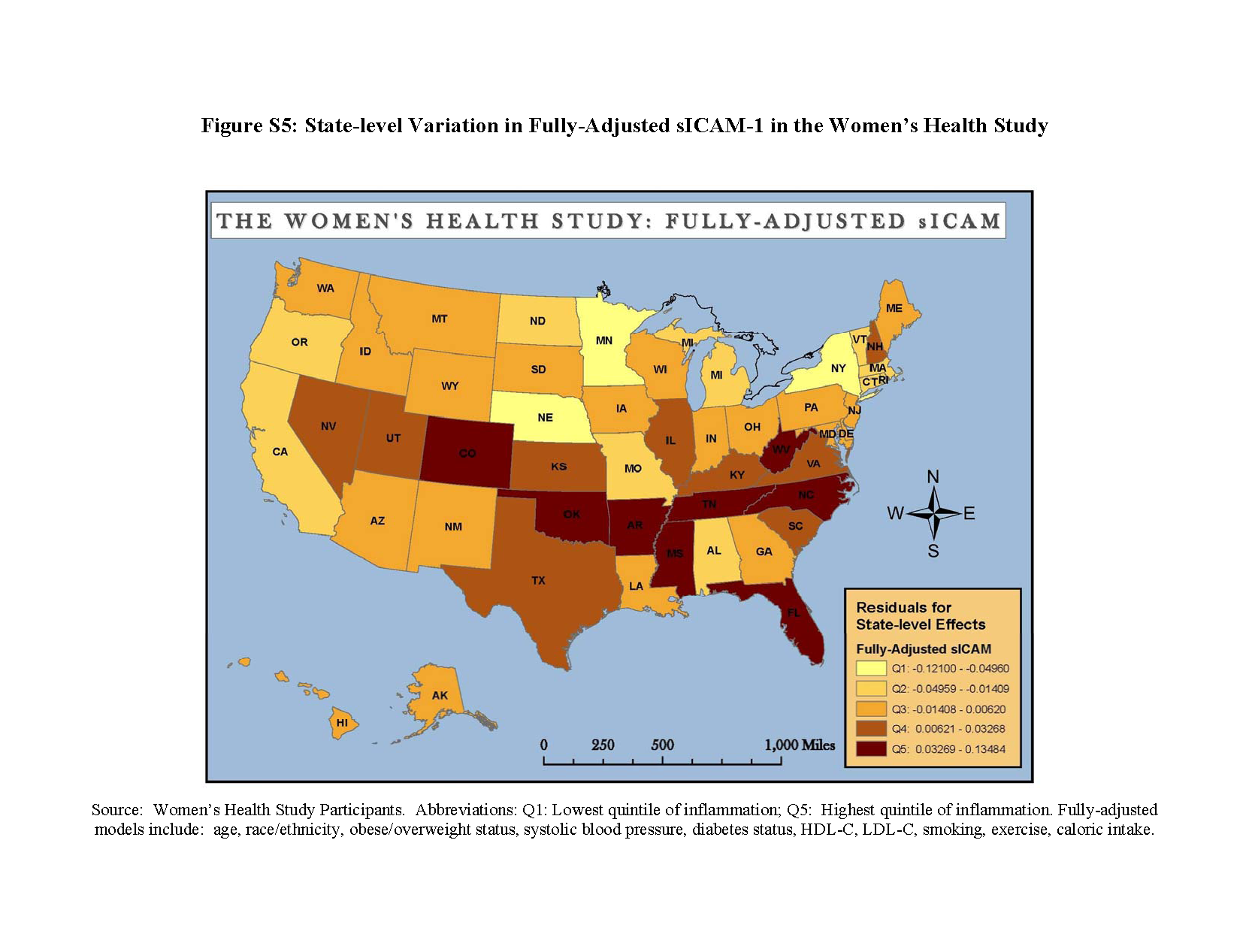

Supplement: Figure S5 — State-level Variation in Fully-Adjusted sICAM-1 in the Women's Health Study. Source: Women's Health Study Participants. Abbreviations: Q1: Lowest quintile of inflammation; Q5: Highest quintile of inflammation. Fully-adjusted models include: age, race/ethnicity, obese/overweight status, systolic blood pressure, diabetes status, HDL-C, LDL-C, smoking, exercise, caloric intake. (TIF) [file pone.0027468.s005.tif]

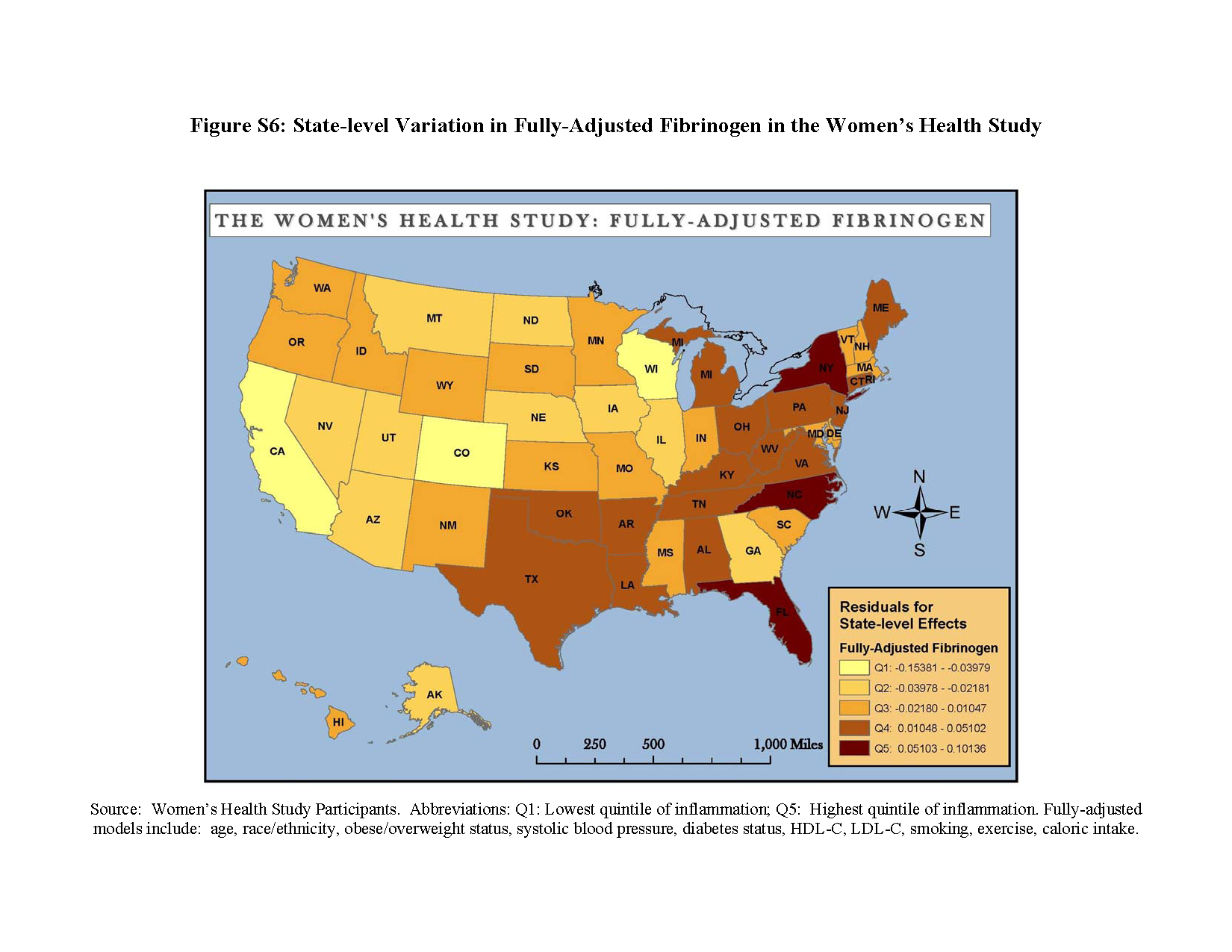

Supplement: Figure S6 — State-level Variation in Fully-Adjusted Fibrinogen in the Women's Health Study. Source: Women's Health Study Participants. Abbreviations: Q1: Lowest quintile of inflammation; Q5: Highest quintile of inflammation. Fully-adjusted models include: age, race/ethnicity, obese/overweight status, systolic blood pressure, diabetes status, HDL-C, LDL-C, smoking, exercise, caloric intake. (TIF) [file pone.0027468.s006.tif]

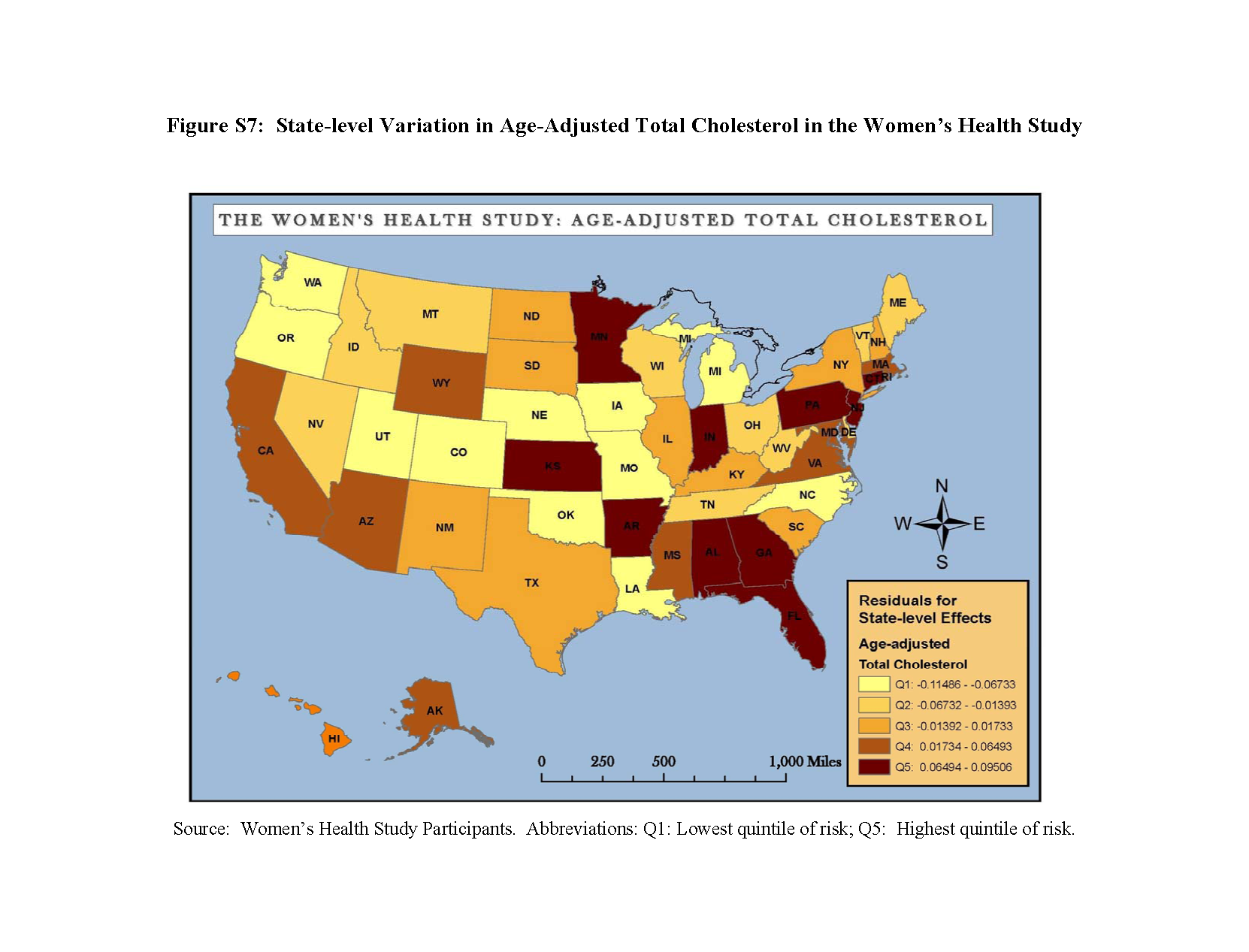

Supplement: Figure S7 — State-level Variation in Age-Adjusted Total Cholesterol in the Women's Health Study. Source: Women's Health Study Participants. Abbreviations: Q1: Lowest quintile of risk; Q5: Highest quintile of risk. (TIF) [file pone.0027468.s007.tif]

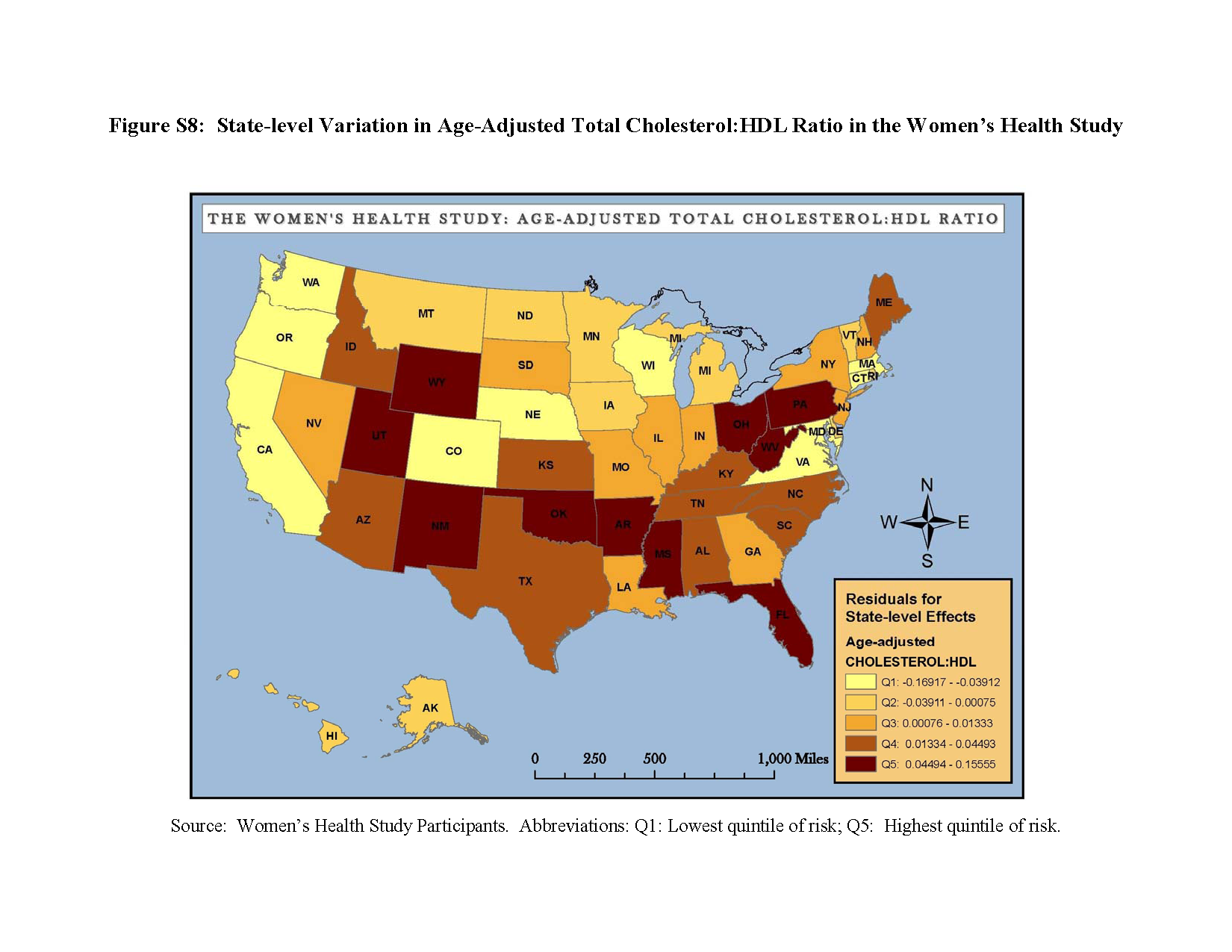

Supplement: Figure S8 — State-level Variation in Age-Adjusted Total Cholesterol:HDL Ratio in the Women's Health Study. Source: Women's Health Study Participants. Abbreviations: Q1: Lowest quintile of risk; Q5: Highest quintile of risk. (TIF) [file pone.0027468.s008.tif]

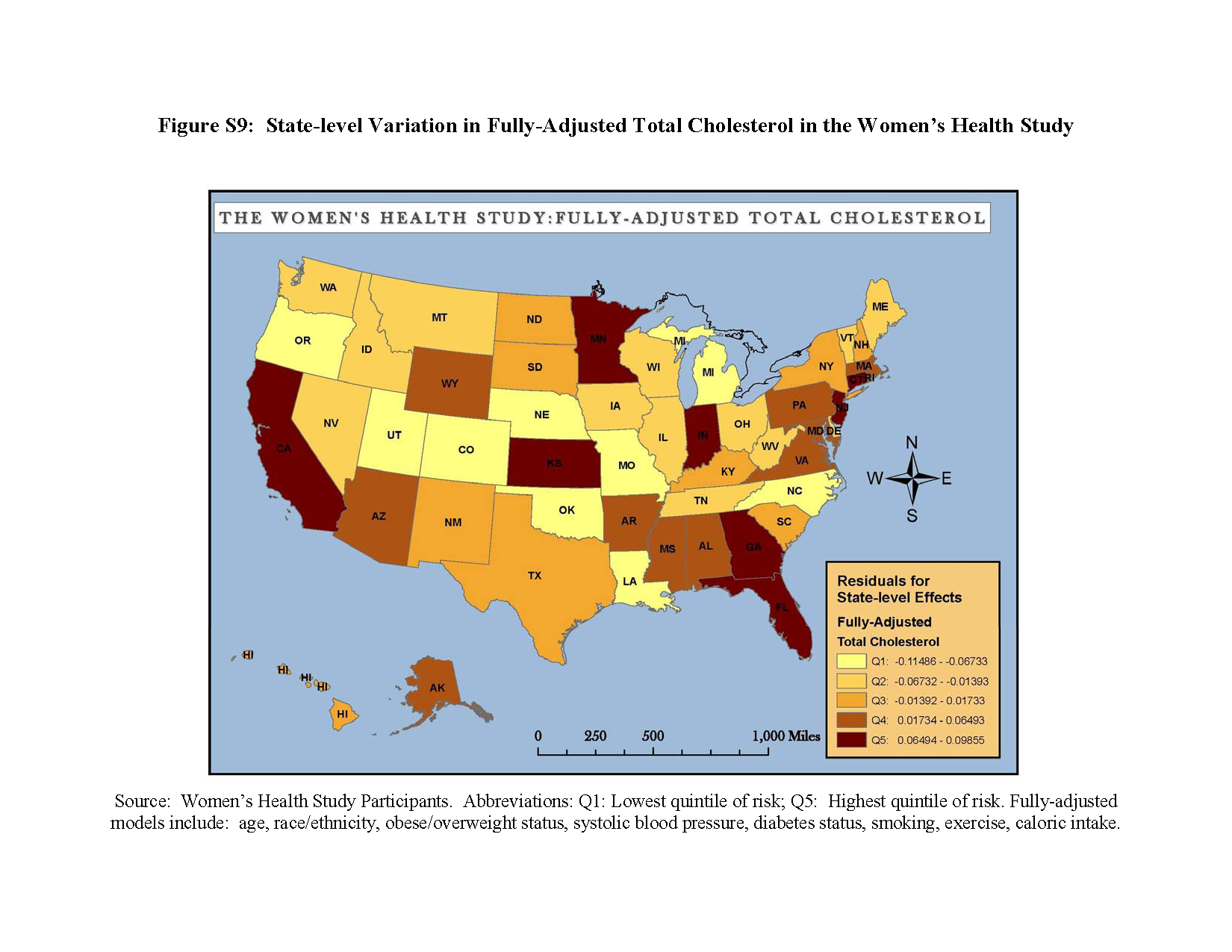

Supplement: Figure S9 — State-level Variation in Fully-Adjusted Total Cholesterol in the Women's Health Study. Source: Women's Health Study Participants. Abbreviations: Q1: Lowest quintile of risk; Q5: Highest quintile of risk. Fully-adjusted models include: age, race/ethnicity, obese/overweight status, systolic blood pressure, diabetes status, smoking, exercise, caloric intake. (TIF) [file pone.0027468.s009.tif]

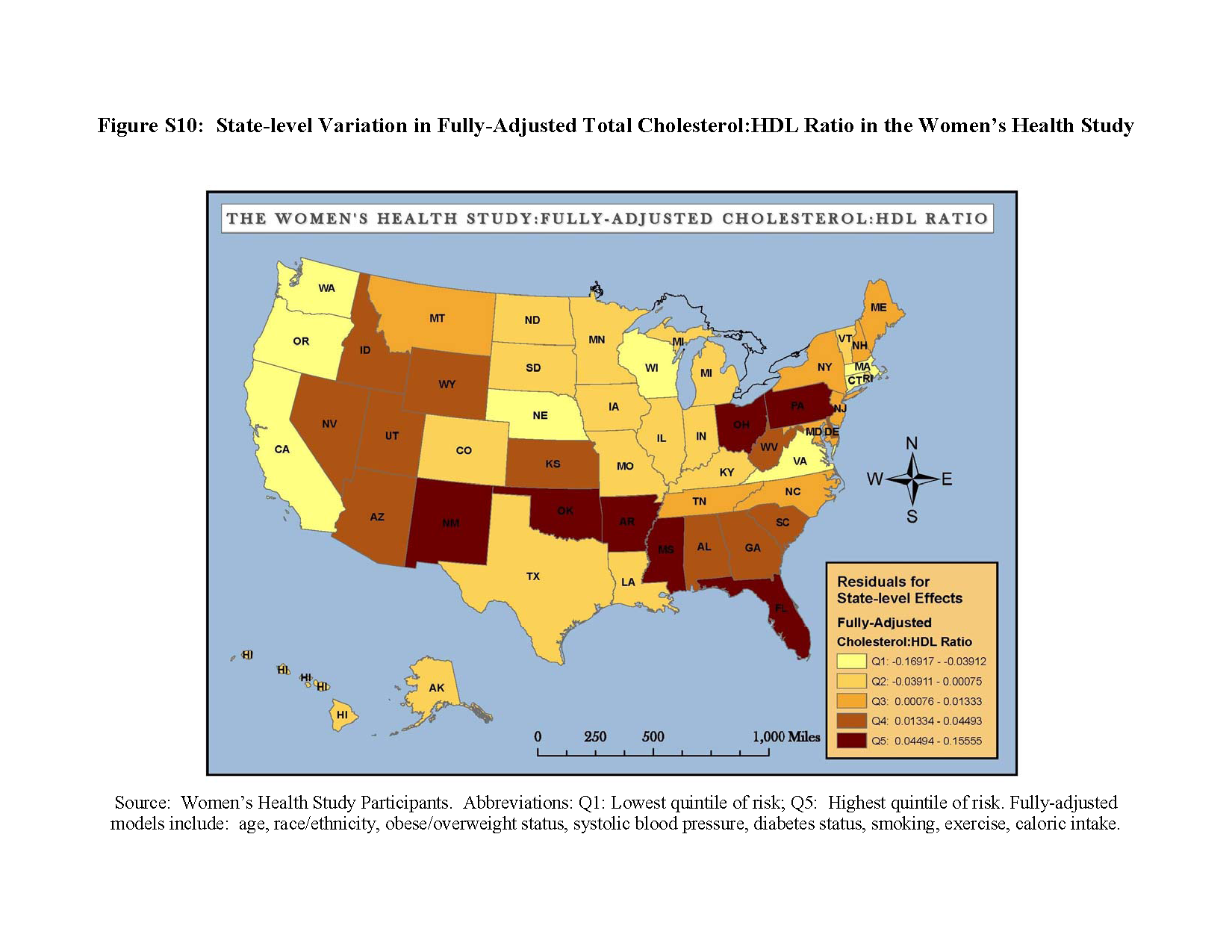

Supplement: Figure S10 — State-level Variation in Fully-Adjusted Total Cholesterol:HDL Ratio in the Women's Health Study. Source: Women's Health Study Participants. Abbreviations: Q1: Lowest quintile of risk; Q5: Highest quintile of risk. Fully-adjusted models include: age, race/ethnicity, obese/overweight status, systolic blood pressure, diabetes status, smoking, exercise, caloric intake. (TIF) [file pone.0027468.s010.tif]

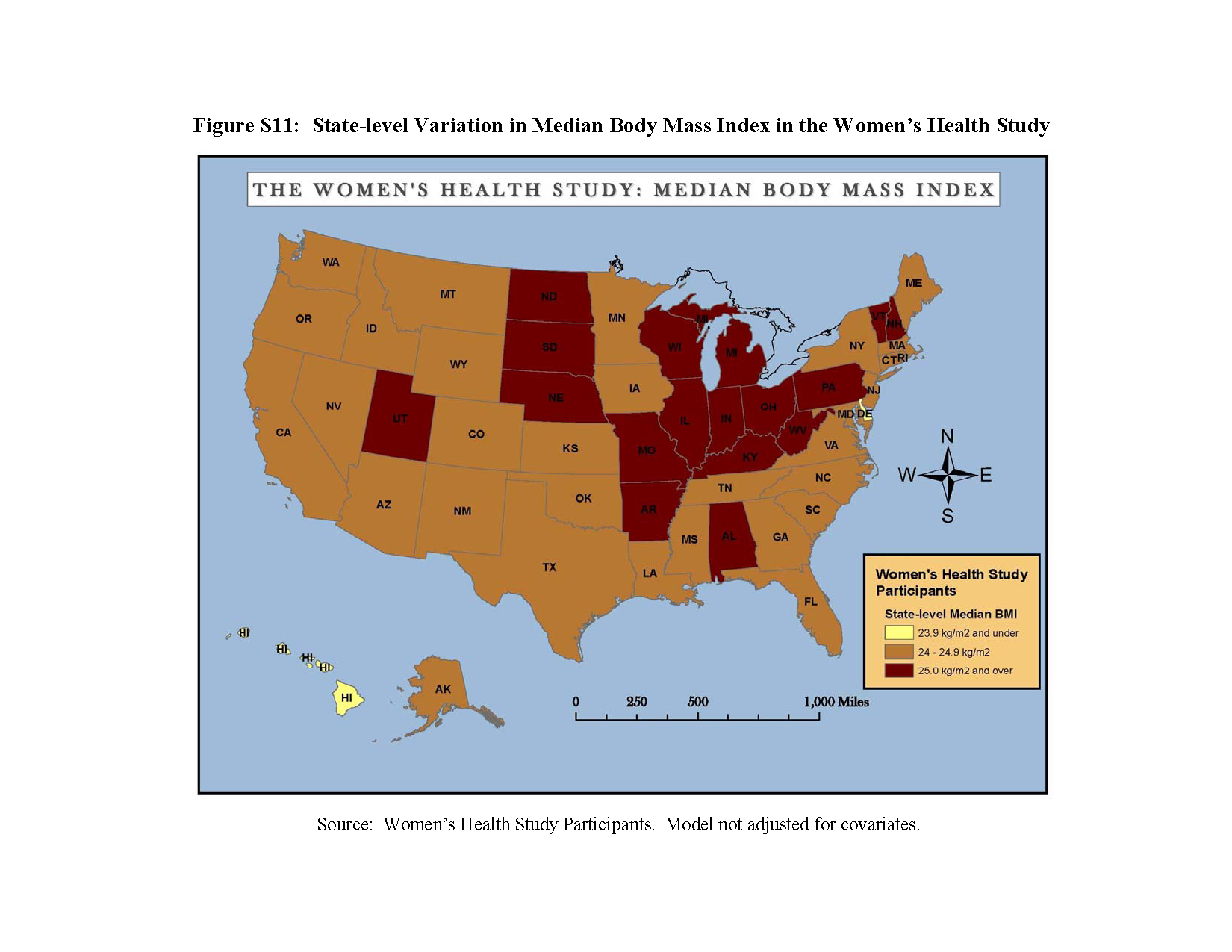

Supplement: Figure S11 — State-level Variation in Median Body Mass Index in the Women's Health Study. Source: Women's Health Study Participants. Model not adjusted for covariates. (TIF) [file pone.0027468.s011.tif]

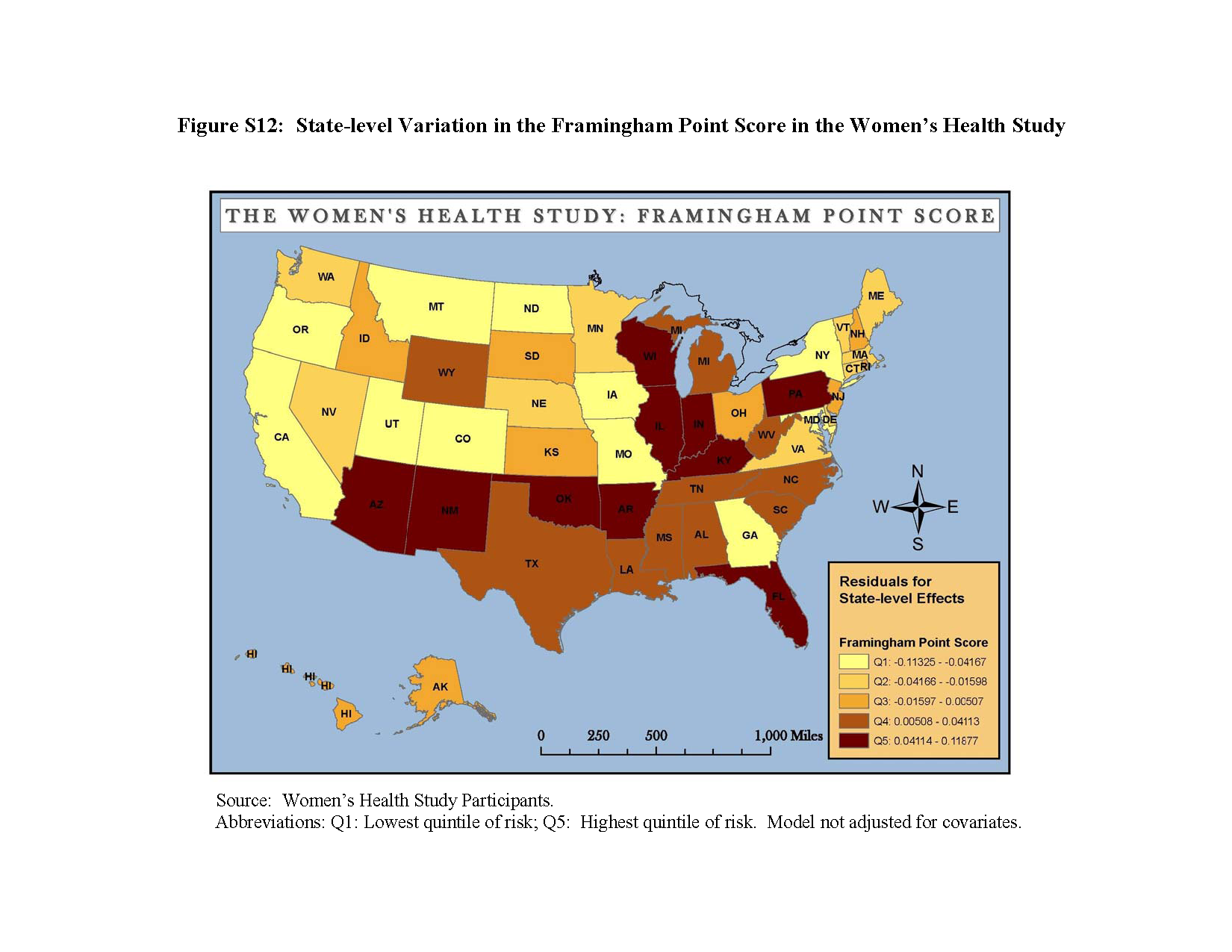

Supplement: Figure S12 — State-level Variation in the Framingham Point Score in the Women's Health Study. Source: Women's Health Study Participants. Abbreviations: Q1: Lowest quintile of risk; Q5: Highest quintile of risk. Model not adjusted for covariates. (TIF) [file pone.0027468.s012.tif]

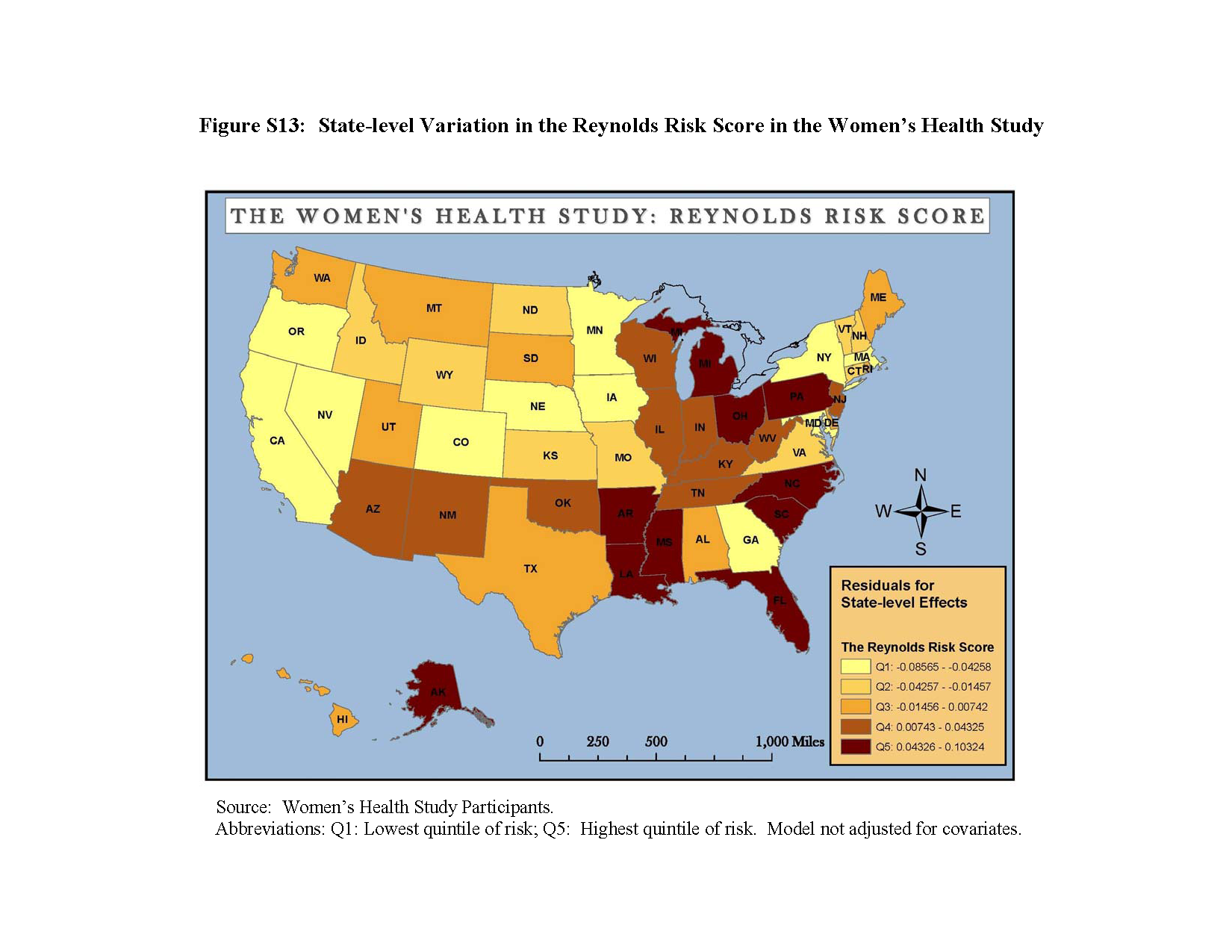

Supplement: Figure S13 — State-level Variation in the Reynolds Risk Score in the Women's Health Study. Source: Women's Health Study Participants. Abbreviations: Q1: Lowest quintile of risk; Q5: Highest quintile of risk. Model not adjusted for covariates. (TIF) [file pone.0027468.s013.tif]
