# Supplementary material for: Geographic Variation in Cardiovascular Inflammation among Healthy Women in the Women's Health Study
Source: PLoS One. 2011 Nov 10;6(11):e27468. doi: 10.1371/journal.pone.0027468 (PMC3213140; doi:10.1371/journal.pone.0027468)
Supplement: Table S1 — State-Level Median Values for Biomarkers of Inflammation among Healthy Women in the Women's Health Study (N = 26,029). *Estimates for District of Columbia and Hawaii based on fewer than 50 participants. (DOC) [file pone.0027468.s014.doc]

**Table S1. State-Level Median Values for Biomarkers of Inflammation among Healthy Women in the Women’s Health Study (N = 26,029)**

|  |  | High-sensitivity | sICAM-1 | Fibrinogen |
| --- | --- | --- | --- | --- |
|  |  | C-Reactive Protein |  |  |
|  |  | Median mg/L | Median ng/mL | Median mg/dL |
|  | N | (standard error) | (standard error) | (standard error) |
| Alabama | 304 | 2.5 (0.35) | 344 (4.4) | 355 (4.9) |
| Alaska | 58 | 2.1 (0.58) | 352 (8.7) | 340 (9.4) |
| Arizona | 610 | 2.3 (0.22) | 341 (3.7) | 344 (3.1) |
| Arkansas | 257 | 2.6 (0.36) | 366 (5.6) | 367 (5.2) |
| California | 2639 | 1.8 (0.11) | 334 (1.5) | 343 (1.5) |
| Colorado | 333 | 2.0 (0.24) | 348 (4.4) | 337 (3.6) |
| Connecticut | 474 | 1.9 (0.20) | 345 (3.7) | 350 (3.7) |
| Delaware | 92 | 1.8 (0.34) | 354 (8.5) | 343 (8.8) |
| District of Columbia* | 25 | 0.9 (0.67) | 320 (14.3) | 331 (16.2) |
| Florida | 1616 | 2.1 (0.18) | 346 (2.3) | 356 (2.1) |
| Georgia | 562 | 2.2 (0.20) | 337 (3.6) | 343 (3.3) |
| Hawaii* | 8 | 1.4 (1.16) | 344 (32.0) | 385 (25.0) |
| Idaho | 194 | 2.1 (0.32) | 340 (5.7) | 345 (5.3) |
| Illinois | 1003 | 2.1 (0.17) | 344 (2.7) | 359 (2.2) |
| Indiana | 631 | 2.2 (0.20) | 341 (3.1) | 353 (3.1) |
| Iowa | 85 | 1.3 (0.79) | 325 (10.3) | 322 (8.9) |
| Kansas | 412 | 2.1 (0.24) | 344 (4.0) | 347 (3.8) |
| Kentucky | 317 | 2.2 (0.31) | 352 (4.7) | 351 (5.0) |
| Louisiana | 282 | 2.5 (0.34) | 354 (3.9) | 355 (4.6) |
| Maine | 318 | 1.8 (0.26) | 344 (4.6) | 354 (4.8) |
| Maryland | 627 | 1.7 (0.18) | 335 (3.3) | 347 (3.2) |
| Massachusetts | 707 | 1.6 (0.13) | 336 (2.9) | 347 (2.9) |
| Michigan | 1046 | 2.0 (0.17) | 343 (2.4) | 354 (2.6) |
| Minnesota | 1075 | 1.8 (0.14) | 338 (2.3) | 347 (2.3) |
| Mississippi | 193 | 2.3 (0.43) | 359 (6.3) | 357 (6.1) |
| Missouri | 701 | 2.1 (0.19) | 341 (3.1) | 352 (2.7) |
| Montana | 142 | 1.8 (0.41) | 343 (6.1) | 340 (6.4) |
| North Carolina | 656 | 2.4 (0.21) | 349 (3.4) | 359 (3.3) |
| North Dakota | 99 | 2.0 (0.46) | 333 (8.0) | 329 (8.7) |
| Nebraska | 236 | 1.8 (0.53) | 333 (5.0) | 348 (5.1) |
| New Hampshire | 225 | 2.0 (0.80) | 350 (5.1) | 352 (4.3) |
| New Jersey | 388 | 1.7 (0.22) | 344 (4.8) | 353 (4.1) |
| New Mexico | 200 | 2.1 (0.41) | 347 (5.2) | 346 (5.3) |
| New York | 1324 | 1.6 (0.15) | 338 (2.2) | 358 (2.2) |
| Nevada | 134 | 1.8 (0.72) | 349 (6.7) | 340 (6.2) |
| Ohio | 911 | 2.0 (0.15) | 344 (2.9) | 356 (2.7) |
| Oklahoma | 318 | 2.4 (0.38) | 348 (4.9) | 361 (4.4) |
| Oregon | 508 | 2.0 (0.17) | 336 (3.5) | 345 (3.4) |
| Pennsylvania | 1043 | 2.1 (0.14) | 352 (2.4) | 358 (2.5) |
| Rhode Island | 218 | 1.7 (0.24) | 332 (5.8) | 353 (5.7) |
| South Carolina | 339 | 2.2 (0.21) | 348 (4.7) | 351 (4.0) |
| South Dakota | 119 | 2.3 (0.32) | 342 (7.5) | 357 (7.6) |
| Tennessee | 419 | 2.4 (0.24) | 354 (4.5) | 359 (3.5) |
| Texas | 1227 | 2.5 (0.20) | 347 (2.5) | 356 (2.4) |
| Utah | 177 | 2.2 (0.29) | 351 (5.2) | 345 (5.2) |
| Vermont | 128 | 1.7 (0.32) | 335 (5.2) | 356 (6.6) |
| Virginia | 570 | 1.9 (0.25) | 344 (3.5) | 352 (3.7) |
| Washington | 641 | 2.1 (0.19) | 340 (3.0) | 346 (2.8) |
| West Virginia | 218 | 2.7 (0.41) | 351 (6.5) | 358 (6.4) |
| Wisconsin | 1148 | 1.8 (0.16) | 345 (2.4) | 346 (2.3) |
| Wyoming | 72 | 2.2 (0.80) | 357 (7.6) | 326 (9.0) |
| Total | 26,029 | 2.0 (0.03) | 343 (0.5) | 351 (0.5) |

*Estimates for District of Columbia and Hawaii based on fewer than 50 participants
