# Supplementary material for: Geographic Variation in Cardiovascular Inflammation among Healthy Women in the Women's Health Study
Source: PLoS One. 2011 Nov 10;6(11):e27468. doi: 10.1371/journal.pone.0027468 (PMC3213140; doi:10.1371/journal.pone.0027468)
Supplement: Table S2 — Fully-Adjusted Multi-Level Linear Regression Models on log(hsCRP): Standardized β Coefficients, 95% Confidence Intervals and Wald Tests (N = 26,029). Source: Women's Health Study (WHS). Abbreviations: log(hsCRP) log (High-Sensitivity C-Reactive Protein); NH (Non-Hispanic ethnicity); HDL-C (high-density lipoprotein cholesterol); LDL-C (low-density lipoprotein cholesterol); BMI (body mass index); ref. (reference category). Standardized β coefficients estimated via multi-level linear regression models adjusted for listed covariates. In our primary analysis race ethnicity is modeled as non-White vs. NH-White due to small numbers. In models with race as a categorical variable, log(hsCRP) is lower among Asian/Pacific Islanders compared to NH-Whites [Std β -0.26; 95% CI -0.35, -0.16; P Value < 0.0001]. Hispanics tended to have higher log(hsCRP) compared to NH-Whites [Std β 0.04; 95% CI -0.07, 0.16; P Value 0.48]. NH-Blacks tended to have higher log(hsCRP) compared to NH-Whites [Std β 0.02; 95% CI -0.06, 0.11; P Value 0.61]. (DOC) [file pone.0027468.s015.doc]

**Table S2. Fully-Adjusted Multi-Level Linear Regression Models on log(hsCRP): Standardized β Coefficients, 95% Confidence Intervals and Wald Tests (N=26,029)**

|  | Standardized β Coefficient | Lower 95% Confidence Interval | Upper 95% Confidence Interval | Wald F test Statistic | *P* Value |
| --- | --- | --- | --- | --- | --- |
| Age, (continuous) | 0.11 | 0.099 | 0.121 | 356.10 | < 0.0001 |
| NH-White | 0.06 | 0.010 | 0.109 | 5.55 | 0.02 |
| Non-White | ref. | ref. | ref. |  |  |
| Obese (BMI ≥ 30) | 0.97 | 0.935 | 1.002 | 1758.33 | < 0.0001 |
| Overweight (25 < BMI < 30 ) | 0.50 | 0.477 | 0.529 |  |  |
| Healthy weight (BMI ≤ 25 ) | ref. | ref. | ref. |  |  |
| Systolic blood pressure category | 0.10 | 0.087 | 0.111 | 261.49 | < 0.0001 |
| Diabetic | 0.32 | 0.253 | 0.382 | 92.64 | < 0.0001 |
| Non-Diabetic | ref. | ref. | ref. |  |  |
| Exercise rarely / never | 0.15 | 0.116 | 0.191 | 28.68 | < 0.0001 |
| Exercise < 1 time/week | 0.13 | 0.088 | 0.169 |  |  |
| Exercise 1-3 times/week | 0.07 | 0.028 | 0.102 |  |  |
| Exercise 4+ times/week | ref. | ref. | ref. |  |  |
| Current smoker | 0.16 | 0.122 | 0.193 | 38.12 | < 0.0001 |
| Past smoker | 0.03 | 0.011 | 0.058 |  |  |
| Never smoke | ref. | ref. | ref. |  |  |
| Daily Caloric intake | 0.005 | -0.006 | 0.015 | 0.68 | 0.41 |
| HDL-C | -0.01 | -0.023 | 0.0001 | 3.75 | 0.05 |
| LDL-C | 0.01 | -0.004 | 0.018 | 1.72 | 0.19 |
|  |  |  |  |  |  |

Source: Women’s Health Study (WHS). Abbreviations: log(hsCRP) log(High-Sensitivity C-Reactive Protein); NH (Non-Hispanic ethnicity); HDL-C (high-density lipoprotein cholesterol); LDL-C (low-density lipoprotein cholesterol); BMI (body mass index); ref. (reference category). Standardized β coefficients estimated via multi-level linear regression models adjusted for listed covariates. In our primary analysis race ethnicity is modeled as non-White vs. NH-White due to small numbers. In models with race as a categorical variable, log(hsCRP) is lower among Asian/Pacific Islanders compared to NH-Whites [Std β -0.26; 95% CI -0.35, -0.16; *P* Value < 0.0001]. Hispanics tended to have higher log(hsCRP) compared to NH-Whites [Std β 0.04; 95% CI -0.07, 0.16; *P* Value 0.48]. NH-Blacks tended to have higher log(hsCRP) compared to NH-Whites [Std β 0.02; 95% CI -0.06, 0.11; *P* Value 0.61].
