# Supplementary material for: Geographic Variation in Cardiovascular Inflammation among Healthy Women in the Women's Health Study
Source: PLoS One. 2011 Nov 10;6(11):e27468. doi: 10.1371/journal.pone.0027468 (PMC3213140; doi:10.1371/journal.pone.0027468)
Supplement: Table S3 — Fully-Adjusted Multi-Level Linear Regression Models on sICAM-1: Standardized β Coefficients, 95% Confidence Intervals and Wald Tests (N = 26,029). Source: Women's Health Study (WHS). Abbreviations: NH (Non-Hispanic ethnicity); HDL-C (high-density lipoprotein cholesterol); LDL-C (low-density lipoprotein cholesterol); BMI (body mass index); ref (reference category). Standardized β coefficients estimated via multi-level linear regression models adjusted for listed covariates. In our primary analysis race ethnicity is modeled as non-White vs. NH-White due to small numbers. In models with race as a categorical variable, sICAM-1 is lower among Asian/Pacific Islanders compared to NH-Whites [Std β -0.31; 95% CI -0.41, -0.22; P Value < 0.0001]. Hispanics tended to have higher sICAM-1 compared to NH-Whites [Std β 0.02; 95% CI -0.10, 0.14; P Value 0.72]. NH-Blacks tended to have lower sICAM-1 values compared to NH-Whites [Std β -0.67; 95% CI -0.76, 0.59; P Value < 0.0001]. (DOC) [file pone.0027468.s016.doc]

**Table S3. Fully-Adjusted Multi-Level Linear Regression Models on sICAM-1: Standardized β Coefficients, 95% Confidence Intervals and Wald Tests (N=26,029)**

|  | Standardized β Coefficient | Lower 95% Confidence Interval | Upper 95% Confidence Interval | Wald F test Statistic | *P* Value |
| --- | --- | --- | --- | --- | --- |
| Age, (continuous) | 0.10 | 0.093 | 0.116 | 316.54 | < 0.0001 |
| NH-White | 0.29 | 0.244 | 0.344 | 133.68 | < 0.0001 |
| Non-White | ref. | ref. | ref. |  |  |
| Obese (BMI ≥ 30) | 0.33 | 0.294 | 0.361 | 180.08 | < 0.0001 |
| Overweight (25 < BMI < 30 ) | 0.10 | 0.070 | 0.122 |  |  |
| Healthy weight (BMI ≤ 25 ) | ref. | ref. | ref. |  |  |
| Systolic blood pressure category | 0.05 | 0.040 | 0.065 | 72.48 | < 0.0001 |
| Diabetic | 0.51 | 0.443 | 0.574 | 232.73 | < 0.0001 |
| Non-Diabetic | ref. | ref. | ref. |  |  |
| Exercise rarely / never | 0.10 | 0.062 | 0.137 | 13.36 | < 0.0001 |
| Exercise < 1 time/week | 0.06 | 0.018 | 0.100 |  |  |
| Exercise 1-3 times/week | 0.03 | -0.009 | 0.067 |  |  |
| Exercise 4+ times/week | ref. | ref. | ref. |  |  |
| Current smoker | 1.04 | 1.004 | 1.075 | 1684.71 | < 0.0001 |
| Past smoker | 0.05 | 0.023 | 0.070 |  |  |
| Never smoke | ref. | ref. | ref. |  |  |
| Daily Caloric intake | 0.01 | 0.0007 | 0.022 | 4.35 | 0.04 |
| HDL-C | -0.12 | -0.135 | -0.112 | 427.46 | < 0.0001 |
| LDL-C | 0.05 | 0.042 | 0.064 | 87.26 | < 0.0001 |
|  |  |  |  |  |  |

Source: Women’s Health Study (WHS). Abbreviations: NH (Non-Hispanic ethnicity); HDL-C (high-density lipoprotein cholesterol); LDL-C (low-density lipoprotein cholesterol); BMI (body mass index); ref (reference category). Standardized β coefficients estimated via multi-level linear regression models adjusted for listed covariates. In our primary analysis race ethnicity is modeled as non-White vs. NH-White due to small numbers. In models with race as a categorical variable, sICAM-1 is lower among Asian/Pacific Islanders compared to NH-Whites [Std β -0.31; 95% CI -0.41, -0.22; *P* Value < 0.0001]. Hispanics tended to have higher sICAM-1 compared to NH-Whites [Std β 0.02; 95% CI -0.10, 0.14; *P* Value 0.72]. NH-Blacks tended to have lower sICAM-1 values compared to NH-Whites [Std β -0.67; 95% CI -0.76, 0.59; *P* Value < 0.0001].
