# Supplementary material for: Geographic Variation in Cardiovascular Inflammation among Healthy Women in the Women's Health Study
Source: PLoS One. 2011 Nov 10;6(11):e27468. doi: 10.1371/journal.pone.0027468 (PMC3213140; doi:10.1371/journal.pone.0027468)
Supplement: Table S4 — Fully-Adjusted Multi-Level Linear Regression Models on Fibrinogen: Standardized β Coefficients, 95% Confidence Intervals and Wald Tests (N = 26,029). Source: Women's Health Study (WHS). Abbreviations: NH (Non-Hispanic ethnicity); HDL-C (high-density lipoprotein cholesterol); LDL-C (low-density lipoprotein cholesterol); BMI (body mass index). Standardized β coefficients estimated via multi-level linear regression models adjusted for listed covariates. In our primary analysis race ethnicity is modeled as non-White vs. NH-White due to small numbers. In models with race as a categorical variable, fibrinogen is higher among Asian/Pacific Islanders compared to NH-White [Std β 0.07; 95% CI -0.03, 0.17; P Value 0.17]. Hispanics tended to have higher fibrinogen levels compared to NH-Whites [Std β 0.09; 95% CI -0.03, 0.21; P Value 0.15]. NH-Blacks tended to have higher fibrinogen levels compared to NH-Whites [Std β 0.42; 95% CI 0.33, 0.50; P Value < 0.0001]. (DOC) [file pone.0027468.s017.doc]

**Table S4. Fully-Adjusted Multi-Level Linear Regression Models on Fibrinogen: Standardized β Coefficients, 95% Confidence Intervals and Wald Tests (N=26,029)**

|  | Standardized β Coefficient | Lower 95% Confidence Interval | Upper 95% Confidence Interval | Wald F test Statistic | *P* Value |
| --- | --- | --- | --- | --- | --- |
| Age, (continuous) | 0.16 | 0.146 | 0.170 | 689.29 | < 0.0001 |
| NH-White | -0.20 | -0.252 | -0.150 | 60.01 | < 0.0001 |
| Non-White | ref. | ref. | ref. |  |  |
| Obese (BMI ≥ 30) | 0.62 | 0.582 | 0.651 | 616.90 | < 0.0001 |
| Overweight (25 < BMI < 30 ) | 0.23 | 0.200 | 0.253 |  |  |
| Healthy weight (BMI ≤ 25 ) | ref. | ref. | ref. |  |  |
| Systolic blood pressure category | 0.02 | 0.089 | 0.034 | 11.34 | 0.001 |
| Diabetic | 0.32 | 0.253 | 0.386 | 88.05 | < 0.0001 |
| Non-Diabetic | ref. | ref. | ref. |  |  |
| Exercise rarely / never | 0.14 | 0.098 | 0.175 | 23.41 | < 0.0001 |
| Exercise < 1 time/week | 0.10 | 0.052 | 0.135 |  |  |
| Exercise 1-3 times/week | 0.04 | 0.005 | 0.082 |  |  |
| Exercise 4+ times/week | ref. | ref. | ref. |  |  |
| Current smoker | 0.27 | 0.231 | 0.304 | 110.00 | < 0.0001 |
| Past smoker | 0.002 | -0.022 | 0.026 |  |  |
| Never smoke | ref. | ref. | ref. |  |  |
| Daily Caloric intake | 0.01 | -0.005 | 0.017 | 1.04 | 0.31 |
| HDL-C | -0.13 | -0.139 | -0.115 | 433.72 | < 0.0001 |
| LDL-C | 0.11 | 0.102 | 0.125 | 385.97 | < 0.0001 |
|  |  |  |  |  |  |

Source: Women’s Health Study (WHS). Abbreviations: NH (Non-Hispanic ethnicity); HDL-C (high-density lipoprotein cholesterol); LDL-C (low-density lipoprotein cholesterol); BMI (body mass index). Standardized β coefficients estimated via multi-level linear regression models adjusted for listed covariates. In our primary analysis race ethnicity is modeled as non-White vs. NH-White due to small numbers. In models with race as a categorical variable, fibrinogen is higher among Asian/Pacific Islanders compared to NH-White [Std β 0.07; 95% CI -0.03, 0.17; *P* Value 0.17]. Hispanics tended to have higher fibrinogen levels compared to NH-Whites [Std β 0.09; 95% CI -0.03, 0.21; *P* Value 0.15]. NH-Blacks tended to have higher fibrinogen levels compared to NH-Whites [Std β 0.42; 95% CI 0.33, 0.50; *P* Value < 0.0001].
